# Comparative pathogenicity of infectious bronchitis virus Massachusetts and Delmarva (DMV/1639) genotypes in laying hens

**DOI:** 10.3389/fvets.2023.1329430

**Published:** 2024-01-19

**Authors:** Muhammad Farooq, Reham M. Abd-Elsalam, Natalya Ratcliff, Mohamed S. H. Hassan, Shahnas M. Najimudeen, Susan C. Cork, Sylvia Checkley, Yan Dong Niu, Mohamed Faizal Abdul-Careem

**Affiliations:** ^1^Health Research Innovation Center, Faculty of Veterinary Medicine, University of Calgary, Calgary, AB, Canada; ^2^Faculty of Veterinary Medicine, Cairo University, Giza, Egypt; ^3^Department of Avian and Rabbit Medicine, Faculty of Veterinary Medicine, Assiut University, Assiut, Egypt

**Keywords:** apoptosis, Delmarva (DMV)/1639 strain, infectious bronchitis virus, Massachusetts (Mass) strain, pathogenesis, laying hen

## Abstract

Infectious bronchitis (IB) is a highly contagious and acute viral disease of chicken caused by the infectious bronchitis virus (IBV) of the family Coronaviridae. Even with extensive vaccination against IB by the poultry industry, the occurrence of new IBV genotypes is a continuous challenge encountered by the global poultry industry. This experiment was designed to compare the pathogenicity of two IBV strains belonging to Massachusetts (Mass) and Delmarva DMV/1639 genotypes. Specific pathogen-free laying hens were challenged during the peak of production (30 weeks), keeping a mock-infected control group. During 21 days of observation following infection, a significant drop in egg production with miss-shaped and soft shells was observed in the DMV/1639 IBV-infected hens only. The DMV/1639 IBV infected group showed prolonged and higher cloacal viral shedding compared with the Mass IBV-infected group. At the end of the study (21 days post-infection), the viral genome loads in the respiratory, urogenital, and immune tissues were significantly higher in the DMV/1639 IBV-infected group compared with the Mass IBV-infected group. Macroscopic lesions such as distorted ova leading to egg peritonitis were observed only in the DMV/1639 IBV-infected group. Moreover, microscopic lesion scores were significantly higher in the lung, kidney, cecal tonsils, and oviduct of the DMV/1639 IBV-infected group compared with the Mass IBV-infected group. Finally, the apoptosis index in the kidney, ovary, magnum, isthmus, and shell gland was significantly higher in the DMV/1639 IBV-infected group compared with the control and Mass-infected groups. This study examined the pathogenicity of two IBV genotypes that are impacting the layer industry in North America.

## 1 Introduction

Infectious bronchitis (IB) is an economically important viral disease of chicken and caused by an avian gamma coronavirus known as infectious bronchitis virus (IBV). Despite extensive vaccination against IB, the emergence of new serotypes and variants that escape from current vaccines has become an increasing concern (1). Being a respiratory virus, IBV initially targets the epithelium of the upper respiratory tract, inducing respiratory clinical manifestations (2). Some IBV strains can reach and replicate in other body systems, such as the urogenital system causing nephritis, reduced egg production, and deformed eggs in layers (3). Infected chickens then become susceptible to secondary infections, most commonly *Mycoplasma* species and other bacteria, that eventually increase the severity of IB in flocks (4).

Some strains of IBV are well known for causing economic losses in the table egg industry by affecting egg production and egg quality (5). In addition to secondary bacterial infections, many other factors such as age of the flock, breed, host immune status, and management can influence the severity of the outcome of IB (6). Several control studies on individual IBV strains have been conducted. For instance, 40% of egg drop was observed in specific pathogen-free (SPF) layers experimentally infected with DMV/1639 IBV (7). The average drop in egg production was 21.67% in layer farms in Western Canada infected with mass IBV. In addition, SPF mature hens, when infected with mass IBV, did not produce regular eggs till 10 days post-infection (dpi) (8). An egg production decline was also observed in hens infected with Mass IBV (9). Similarly, Ark strain of IBV declines egg production up to 15% (10). Layers experimentally infected with D1466 IBV strain dropped 16% of eggs on 10–12 dpi, and the production did not recover till the end of experiment (11).

Tissue tropism and pathogenicity of IBV vary among different strains. A widely distributed strain of IBV, the QX-like genotype, is among one of the known strains for causing cystic lesions in the reproductive tract of young chicks (1). Moreover, DMV/1639 strain of IBV causes cystic oviduct in specific pathogen-free (SPF) chickens when infected at young age (2, 3). Infection with the M41 strain of IBV caused severe tracheal ciliary damage in susceptible chickens (4). Furthermore, Mass and T strains of IBV were virulent and caused lesions in the developing oviduct, whereas Conn and Iowa 609 strains did not produce pathology in the immature oviduct (5). Other IBV isolates such as DMV/1639/11 and PA/9579A/10 are determined to be nephropathogenic in susceptible chickens, yielding virus re-isolations from the kidney and inducing characteristic interstitial nephritis microscopic lesions (6).

The programmed cell death that results from the activation of self-destruction intracellular biochemical pathway is called apoptosis (12). The process of apoptosis is activated by a family of cysteine protease (caspases) via extrinsic and intrinsic signaling molecules, depending on the origin of the stimuli (13). Virus-induced apoptosis results in tissue damage and directly interfere with the viral replication in the cells (14). *In vitro* studies have shown that IBV leads to apoptosis in African green monkey kidney cells (Vero), human cells, such as Huh7, and chicken fibroblast (DF1) cells (15, 16). Previously, it has also been shown that IBV is capable of leading to apoptosis *in vitro* in chicken macrophage HD 11 cells (17). Chhabra et al. investigated that IBV 885 and QX strains induce greater apoptosis in chicken embryo kidney cells than M41, whereas M41 causes greater apoptosis index in tracheal organ cultures compared with 885 and QX IBV strains (18), and this *in vitro* and *ex vivo* study provided evidence for possible difference in apoptosis rate due to difference in IBV strains. *In vivo*, it has been shown that Mass IBV induces apoptosis in the trachea, lungs, kidney, and BF early during the infection 12 h to 7 days following infection (19). However, the information is scarce to indicate if the induction of apoptosis in tissues is IBV strain-dependent *in vivo*. Relevant to IBV infection, it has been shown that both intrinsic and extrinsic mechanisms are involved in IBV-induced apoptosis (17, 20).

Studies, that are directed to investigate pathogenesis of specific IBV strains, will clarify the pathogenicity of the viral strains, leading to inclusion of these IBV strains when developing new vaccines (21). Recently, the DMV/1639 strain of IBV was identified as a virulent circulating variant in the layer flocks of Eastern Canada (7). IBV strain, Mass (15AB-01), was isolated from the layer flocks in Alberta (AB), with a background of decreased egg production, producing egg shell abnormalities (8). Based on the spike (S)1 gene, DMV/1639 strain of IBV is only 97% similar to original DMV1639 strain described in 2011 in USA (22), and 15AB-01 Mass IBV strain is only 95.5% similar to M41 IBV strain (23). When we compare the S1 gene sequence between DMV1639 and 15AB-01 Mass IBV, the similarity is only 78%. Based on the whole genome sequence, DMV/1639 strain of IBV is 93.7% similar to Connecticut (Conn) vaccine strain (22), and 15AB-01 Mass IBV strain is 92.8% similar to M41 IBV (8). When we compare the whole genome sequence between DMV1639 and 15AB-01 Mass IBV, they are only 98.59% similar. The aim of the current research was to study the comparative pathogenicity and pathogenesis of two IBV strains DMV/1639 and Mass in laying hens, with focus on the ability of the IBV to induce apoptosis in infected cells.

## 2 Materials and methods

### 2.1 Viral strains

The DMV strain (Canadian DMV/1639) of IBV used in this study was isolated from a layer flock in Eastern Canada, with a history of dropped egg production in 2017 (22). The Mass IBV strain (15AB-01) was isolated from chickens with a history of egg shell abnormalities in Western Canada (8). These two IBV strains were titrated in SPF embryonated chicken eggs, aliquoted, and stored at −80°C. The use of eggs and chickens and the described animal-related procedures were approved by the Veterinary Science Animal Care Committee of the University of Calgary (Protocol number: AC19-0011).

### 2.2 Chickens

The SPF White Leghorn hens (24 weeks old) were obtained from the Canadian Food Inspection Agency (CFIA), Ottawa, Canada. The hens (*n* = 36) were maintained in the Veterinary Science Research Station (VSRS) at the Spy Hill Campus of the University of Calgary. The hens were allowed to acclimatize and reach the peak laying period before infection. The feed, water, light, temperature, and humidity were adjusted according to the management procedures advised for White Leghorn mature laying hens.

### 2.3 Experimental design

#### 2.3.1 IBV and mock infection

The hens were randomly equally allocated into three groups (*n* = 12 hens per group) and housed in three separate negative pressure rooms with predetermined directed movement of the staff. The hens in the infected group were inoculated via intratracheal and oculo-nasal route with 1 × 10^6^ embryo infectious dose (EID)_50_ of DMV/1639 IBV and Mass IBV. The control group was given phosphate buffered saline (PBS), having pH 7.4, through the same route.

#### 2.3.2 Clinical observations and swab, blood, and egg collection

Hens in all three groups were observed twice daily (morning, evening) for clinical signs. The clinical signs were scored on a scale of 0 to 3 from no clinical signs to severe signs as illustrated previously (24). In brief, each of general outcomes such as depression, ruffled feathers, and dropped wings was scored 1. The specific signs of mild respiratory scores such as increased respiration with closed beak as 1, and moderate respiratory signs such as increased respiration with open mouth breathing, coughing, watery eyes, and nasal discharge as 2, and severe respiratory signs such as marked gasping as 3. The total aggregate clinical score of 5 for a single bird was the humane end point as per the guidelines of Canadian Council on Animal Care (CCAC). Oropharyngeal (OP) and cloacal (CL) swabs were collected on 3, 10, 17, and 21 dpi using the Puritan^®^ virus transport medium (Puritan Medical Products Co., Guilford, ME, United States). The samples were transported on ice and stored at −80°C till further processing. In addition, all the hens were bled on 10 and 21 dpi to collect 1 mL of blood from the wing vein of the bird in plain tubes which were kept at 4°C overnight. Serum was separated by centrifugation at 1,503 g (4,000 rounds per minute or rpm) for 10 min, aliquoted in 100 μL microcentrifuge tubes, and stored at −20°C. Eggs from all three groups were collected once daily. The eggs were weighed with a sensitive weighing scale (0.01 g LCD Mini Precision). Length, width, and thick albumin height of each egg were measured with vernier caliper (Digital LCD Caliper Model H-7352).

#### 2.3.3 Post-mortem examination and sample collection

On 21 dpi, all the animals were anesthetized with isoflurane and then euthanized through cervical dislocation (25). Samples from the trachea, lung, kidney, spleen, cecal tonsils (CT), ovary, and oviduct were collected in RNA Save^®^ (Biological Industries, Beit Haemek, Israel). Furthermore, segments of the trachea, lung, kidney, CT, magnum, isthmus, and shell gland were also collected in 10% neutral buffered formalin (VWR International, Edmonton, AB, Canada) for histopathology and immunohistochemistry analyses. The tissues in RNA Save were frozen at −80°C until further processing.

### 2.4 Techniques

#### 2.4.1 Quantification of IBV genome load

Total ribonucleic acid (RNA) extraction was performed on the tissue and swab samples using the Trizol^®^ reagent (Invitrogen Canada Inc., Burlington, ON, Canada), following the manufacturers guidelines. The RNA concentration and quality of the extracted RNA were determined by using Nanodrop1000 spectrophotometer (Thermo Scientific, Wilmington, DE, United States), based on the wavelength absorbance of 260/280 nm. In total, 2 μg of RNA of each swab and tissue sample was used to synthesize complimentary deoxyribonucleic acid (cDNA) using High-Capacity cDNA Reverse Transcription Kit (Invitrogen Life Technologies, Carlsbad, CA, United States), according to the manufacturer protocol. The IBV genomes in swab and tissue samples were quantified with SYBR green-based quantitative polymerase chain reaction (qPCR) assay using nucleocapsid gene (N) primers as previously described (26). In-house prepared IBV N-gene plasmid was used to generate the standard curve using 10-fold serial dilutions. All the assays were performed with Fast SYBR^®^ Green Master Mix (Quntabio^®^, Beverly, MA, United States) in 20 μL of reaction volume. The reaction was performed in 96-well PCR plates with 10 μL of SYBR Green Master Mix, 100 ng of respective cDNA template in 2 μL, 0.5 μL each of forward and reverse primers, and 7 μL molecular grade water. The conditions set for thermocycling were 95°C for 20 s (s), followed by 40 cycles of amplification, extension at 95°C for 3 s, and 60°C for 30 s; melting curve analysis was performed at 95°C for 10 s (Segment 1), 65°C for 5 s (Segment 2), and 9°C for 5 s (Segment 3). Fluorescent acquisition was performed at 60°C for 30 s.

#### 2.4.2 Enzyme-linked immunosorbent assay

Antibodies from serum and oviduct washes were analyzed with commercial ELISA kit (IDEXX Laboratories, Inc., Westbrook, ME, United States). Manufacturer protocol and formula were followed for the quantification of antibody titers by considering the titers >396 (cutoff value) as positive.

#### 2.4.3 Histopathology

The tissues (trachea, lung, kidney, spleen, cecal tonsils, ovary, and oviduct) were fixed in 10% neutral buffered formalin, sectioned at 5 μm thickness, and stained with hematoxylin and eosin (H&E) at the Diagnostic Services Unit (DSU) of the University of Calgary. All the slides were examined using light microscopy (Olympus BX51, Center Valley, PA, United States) for lesion scoring attributable to IBV infection. Lesion scorning for the trachea, lung, kidney, and oviduct was performed according to previously published methods (21) with some modification. In brief, lesions such as loss of epithelium, degeneration, necrosis, depletion of mucus glands, and lymphoplasmocytic infiltration were given a score as follows: normal (0); mild (1); moderate (2); and severe (3) (27). Tracheal mucosal thickness was measured using Image J analyzer (National Institute of Health, Bethesda, Maryland, United States). The ventral tracheal mucosa was digitally photographed at 200× magnification power, and then, the total thickness of the mucosa was measured.

#### 2.4.4 Immunohistochemistry

An immunohistochemical technique was performed following the methods described previously (28). The 5 μm thick sections were deparaffinized in two changes of xylene and then rehydrated in alcohol. The endogenous peroxidase activity was blocked by incubating sections in 3% H_2_O_2_ solution in methanol at room temperature for 10 min. The heat antigenic retrieval method was performed to unmask the antigenic epitopes using 10 mM citrate buffer at pH 6.0 (29). The tissue sections were incubated in blocking buffer (2.5% horse serum) for 1 h at room temperature. The primary mouse anti-IBV antibody (Novus Biological, Bio-Techne, Toronto, ON, Canada) diluted (1,400) in 2.5% horse serum was added to the tissue section and incubated at 4°C overnight in a humidified chamber. The tissue sections were incubated with goat anti-mouse secondary antibody (DK-2594, Vector Laboratories Inc., Burlingame, CA, United States) for 30 min. Antibody binding was detected by ABC peroxidase kit (Vector Laboratories Inc., Burlingame, CA, United States) and DAB (3, 3’-Diaminobenzidine) substrate (Vector Laboratories Inc., Burlingame, CA, United States) solution. The sections were counterstained with Gill’s Hematoxylin (Vector Laboratories Inc., Burlingame, CA, United States) and then cover slipped with mounting media Vectashield^®^ 4′, 6-diamidino-2-phenylindole (DAPI, Vector Laboratories Inc., Burlingame, CA, United States).

#### 2.4.5 Terminal deoxynucleotidyl transferase dUTP nick end labeling for apoptosis detection In tissue sections

Apoptosis was detected in the trachea, lung, kidney, ovary, and oviducts using a TUNEL assay kit, following the manufacturer’s procedures (Promega, Madison, Wisconsin, United States) and previously published protocol (30). In brief, paraffin-embedded tissue sections were dewaxed in xylene, rehydrated in different grades of alcohols, and then fixed with 4% paraformaldehyde in PBS for 15 min. The tissue sections were permeabilized by adding 100 μL of a 20 μg/mL proteinase K solution for 30 min at room temperature. Equilibration buffer was added to tissue sections for 10 min. The tissue sections were incubated with a Terminal Deoxynucleotidyl Transferase (TdT) reaction mix for 60 min at 37°C in a humidified chamber. The reaction was stopped by immersing the slides in 2× saline sodium citrate for 15 min. The endogenous peroxidase activity was blocked by incubating sections in 3% H_2_O_2_ solution at room temperature for 5 min. The tissue sections were incubated with streptavidin horseradish peroxidase (diluted 1:500 in PBS) for 30 min at room temperature, and then, the reaction was visualized by adding DAB solution till a light brown background appears (3–5 min depending on the organ type and section thickness). The tissue sections were counterstained with Gill’s Hematoxylin and then mounted with a coverslip. The apoptosis index was calculated as the average number of TUNEL-positive apoptotic cells in five different fields per organ under the 20× objective. The mean apoptotic index represents the average of the apoptotic index from randomly selected six birds per group.

#### 2.4.6 Statistical analysis

A two-way ANOVA test, followed by Tukey’s multiple comparison test, was used for clinical scores and egg measurement data. Egg production among the groups was compared with a 2 × 3 chi-square test. IBV genome loads in OP and CL swabs and tissues were analyzed through two-way ANOVA test, followed by Sidak’s multiple comparison test. A one-way ANOVA test, followed by Tukey’s multiple comparison test, was used to analyze the data of antibody titers. Finally, Kruskal–Wallis test and Dunn’s multiple comparison test were applied for pathological lesion scoring and apoptosis scoring. Assumptions of the statistical tests were assessed and met prior to proceeding with analysis. GraphPad Prism 9.4.1 software (GraphPad Software, San Diego, CA, United States) was used for the statistical analysis and figure generation.

## 3 Results

### 3.1 Clinical manifestation

**Figure 1:**
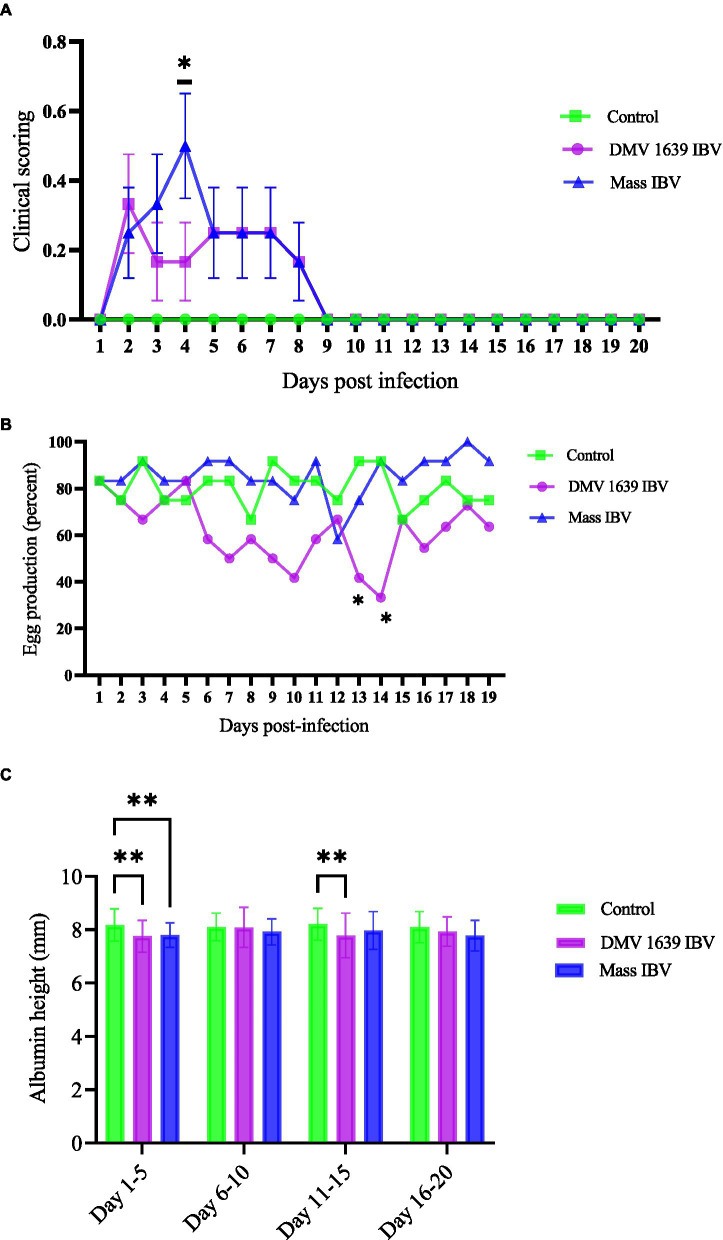
Clinical score, egg production, and thick albumin height. **(A)** Mean clinical scores ± SEM of IBV infected and control hens: The x-axis shows clinical scoring on the scale of 0 to 5 from the scoring sheet and the y-axis shows days post-infection. **(B)** The mean percentage of daily egg production of IBV-infected and control hens with differences analyzed using a 2 × 3 chi-square test. **(C)** The mean albumin height ± SEM was analyzed with a two-way ANOVA. The x-axis shows albumin height in millimeters measured with a digital vernier caliper of 0.01 mm precision, and the y-axis shows the pooled days (5 together) post-infection. Statistical significance was assessed at *p < 0.05, **p < 0.01.

Following IBV infection, hens from all groups were observed twice daily for the development of clinical signs till 21 dpi. No clinical signs were observed in the control group. In contrast, hens in both infected groups (DMV/1639 and Mass IBV) started to show clinical signs, such as ruffled feather and a mild increase in respiration from 2 dpi. In the Mass IBV-infected group, one bird showed moderate respiratory signs (increased respiration, constant open-beak breathing). The mean clinical score of the Mass IBV-infected group was significantly higher at 4 dpi compared with the control group (*p <* 0.05) (Figure 1A). From 7 dpi onward, all the hens in the infected groups started to become normal; we did not observe clinical manifestations in the IBV-infected groups.

### 3.2 Egg production and measurements

During their peak egg production (80–100%), the hens in the two groups were infected with IBV (DMV/1639, Mass), and one group was kept as control. Egg production in the control and Mass IBV-infected groups did not decline, except on 12 dpi, the Mass IBV-infected group produced approximately 55% eggs. Egg production of the DMV/1639 IBV-infected group significantly dropped (*p* < 0.05). In the DMV/1639 IBV-infected group, on 13 and 14 dpi, there were 40 and 30% drop in egg production, respectively (Figure 1B).

Although egg parameters such as weight, length, width, and thick albumin height were measured till the 20 dpi, only the thick albumin (immediately surrounding the yolk) height was found to be significantly different among the groups. The thick albumin height of both DMV/1639 and Mass IBV infected groups was significantly less (*p* < 0.05) than the control group from 1 to 5 days eggs pooled data, while from 11 to 15 days only the DMV/1639 IBV infected group had eggs with significantly thinner albumin (*p* < 0.05) than that form the MASS and uninfected control group (Figure 1C).

### 3.3 IBV genome loads in swab samples

**Figure 2:**
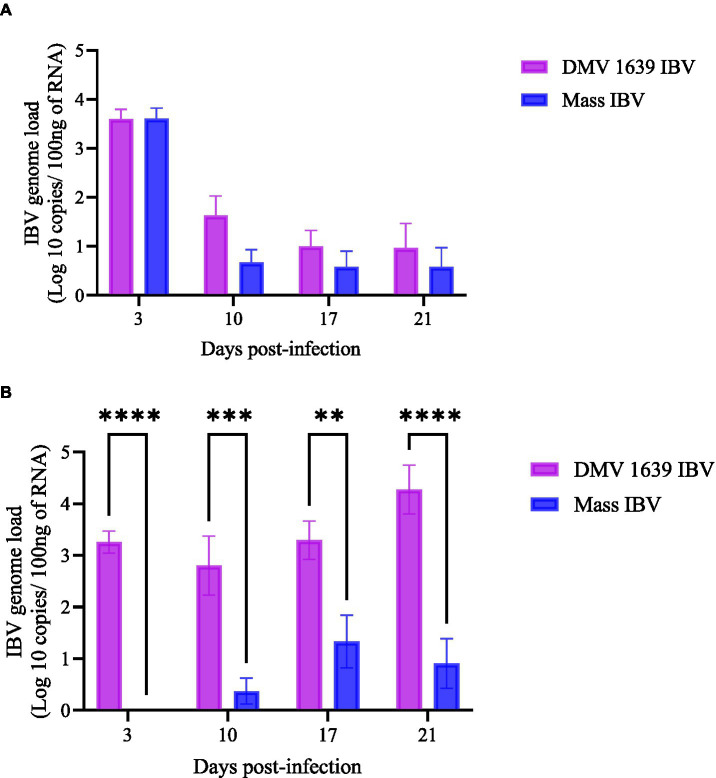
IBV genome loads in swab samples collected at 3, 10, 17, and 21 dpi. **(A)** The mean IBV genome load of DMV/1639 IBV-infected and Mass IBV-infected hens in OP swabs. **(B)** The mean IBV genome load of DMV/1639-infected and Mass IBV-infected hens in CL swabs. The IBV copies were measured per 100 ng of the extracted RNA. The error bars show ± SEM analyzed with a two-way ANOVA, followed by Sidak’s multiple comparison test, and statistical significance was considered at **p < 0.01, ***p < 0.001, and ****p < 0.0001.

At 3, 10, 17, and 21 dpi, no IBV genome was quantified in both OP or CL swab samples of the control group. In the OP swab, the IBV genome load was the highest on 3 dpi in both IBV-infected groups. Although IBV shedding gradually declined by 21 dpi, 3 birds from the DMV IBV-infected group and 2 birds from the Mass IBV-infected group continued with shedding. There was no significant difference in the OP IBV genome load between the two IBV-infected groups (Figures 2A, *p* > 0.05). In contrast, the IBV genome loads in the CL swabs of the DMV IBV-infected group were significantly higher (*p* < 0.05) at 3, 10, 17, and 21 dpi than that observed in the Mass IBV-infected group (Figure 2B). The CL route viral shedding of the DMV/1639 IBV group continuously increased from 3 dpi to the end of the experiment (21 dpi), while in the Mass IBV-infected group, only 2–3 hens started CL viral shedding at 10 dpi, peaked at 17 dpi, and then dropped by 21 dpi (Figure 2).

### 3.4 Anti-IBV antibody titers

**Figure 3:**
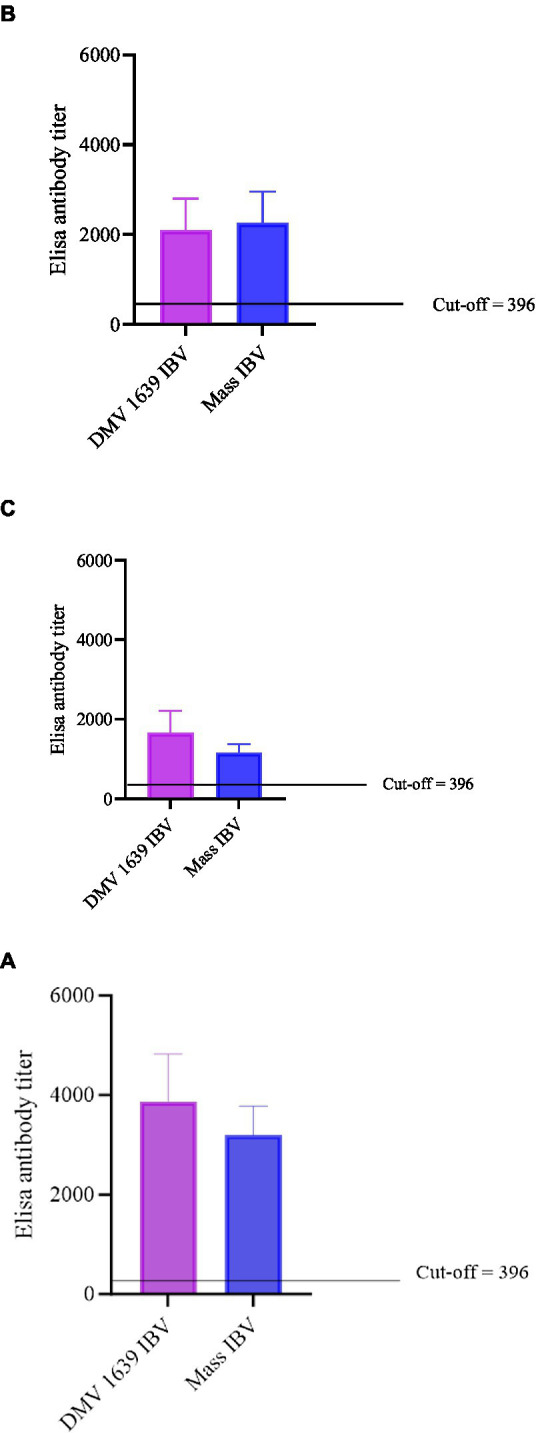
Anti-IBV antibody titers in serum and oviduct washes of IBV-infected and control hens. **(A)** Anti-IBV antibody titers in serum at 10 dpi. **(B)** Anti-IBV antibody titers in serum at 21 dpi. **(C)** Anti-IBV antibody ELISA titers in oviduct washes collected at 21 dpi. Mean titers of all the groups were analyzed with one-way ANOVA followed by Tukey’s multiple comparison test. The error bars represent ±SEM, and statistical significance is considered at *p < 0.05, **p < 0.01, and ***p < 0.001.

The anti-IBV antibody concentrations in serum and reproductive tract washes are presented in Figure 3. At 10 and 21 dpi, no anti-IBV antibody titer was quantifiable in the serum and reproductive tract washes of the control group. At 10 and 21 dpi, anti-IBV antibody titer in the serum and oviduct washes of both the IBV-infected groups was significantly higher (*p* < 0.05) than the control group. At 10 and 21 dpi, no statistical difference (*p* > 0.05) was noted between the serum and oviduct wash anti-IBV antibody titers of the DMV/1639 IBV-infected and Mass IBV-infected groups.

### 3.5 IBV replication in the tissues

**Figure 4:**
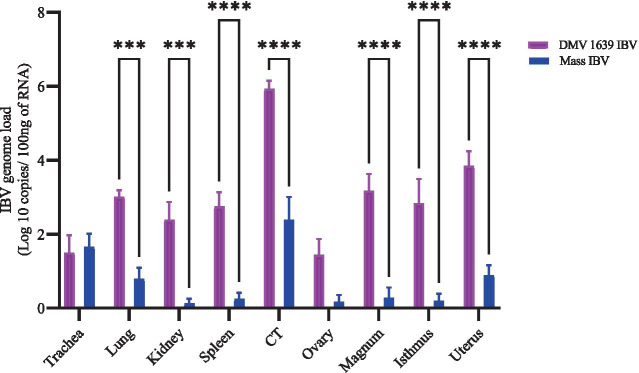
IBV genome loads in tissues collected at 21 dpi following infection with DMV/1639 IBV and Mass IBV. The error bars represent ± SEM analyzed. The IBV genome copies were quantified per 100 ng of the extracted RNA. The IBV genome loads among tissues were compared using a two-way ANOVA, followed by Sidak’s multiple comparison test, and statistical significance was considered at ***p < 0.001 and ****p < 0.0001.

IBV genome load in various tissues was measured using RT-qPCR (Figure 4). At 21 dpi, the control group showed no quantifiable IBV genome in any tissue. In contrast, the DMV IBV-infected group displayed a significantly higher IBV genome load in several tissues, including the lung, kidney, spleen, CT, magnum, isthmus, and shell gland, compared with the Mass IBV-infected group (*p* < 0.05). The highest genome load detected was in the CT of the DMV IBV-infected group. The IBV genome loads in the trachea and ovary were not different significantly (*p* > 0.05).

### 3.6 Immunohistochemistry

**Figure 5:**
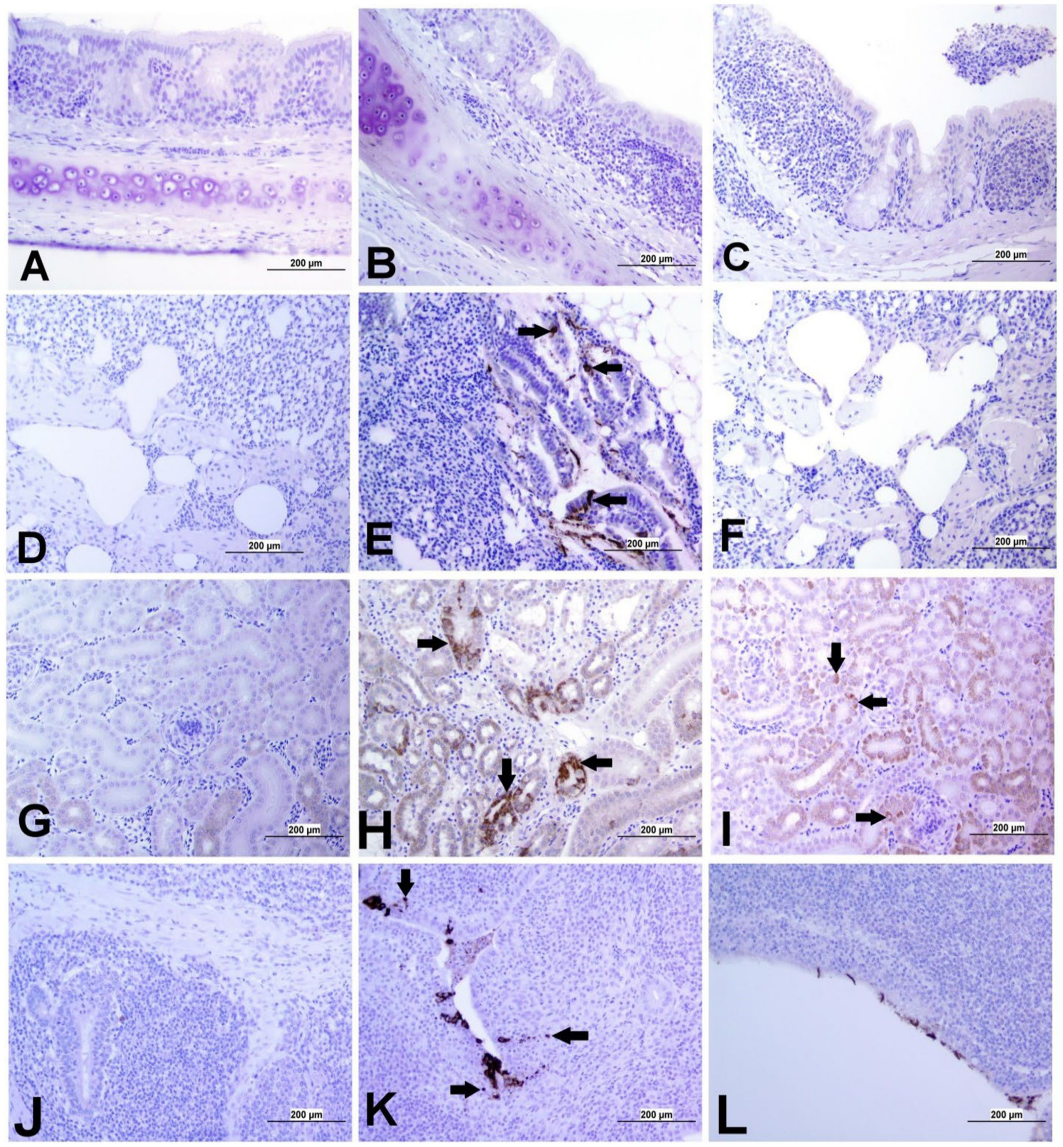
Representative images captured following immunohistochemical analysis of IBV antigens in different tissues at 21 dpi. **(A)** Trachea of control group, **(B)** Trachea of DMV/1639 IBV-infected group, and **(C)** Trachea of Mass IBV-infected group, showing no immunopositive reaction. **(D)** Lung of control group; **(E)** Lung of DMV/1639 IBV-infected group showing immunopositive staining in epithelium lining of bronchi (arrow). **(F)** Lung of the Mass IBV-infected group showing no immunopositive reaction. **(G)** Kidney of control group; **(H)** Kidney of the DMV/1639 IBV-infected group showing strong immunopositive reaction in renal tubules (arrow). **(I)** Kidney of the Mass IBV-infected group showing weak immunopositive reaction in renal tubules (arrow). **(J)** CT of control group; **(K)** CT of the DMV/1639 IBV-infected group showing strong immunopositive reaction in lymphoepithelium and sub-epithelium inflammatory cells (arrow); **(L)** CT of the Mass IBV-infected group with immunopositive reaction in the epithelium.

**Figure 6:**
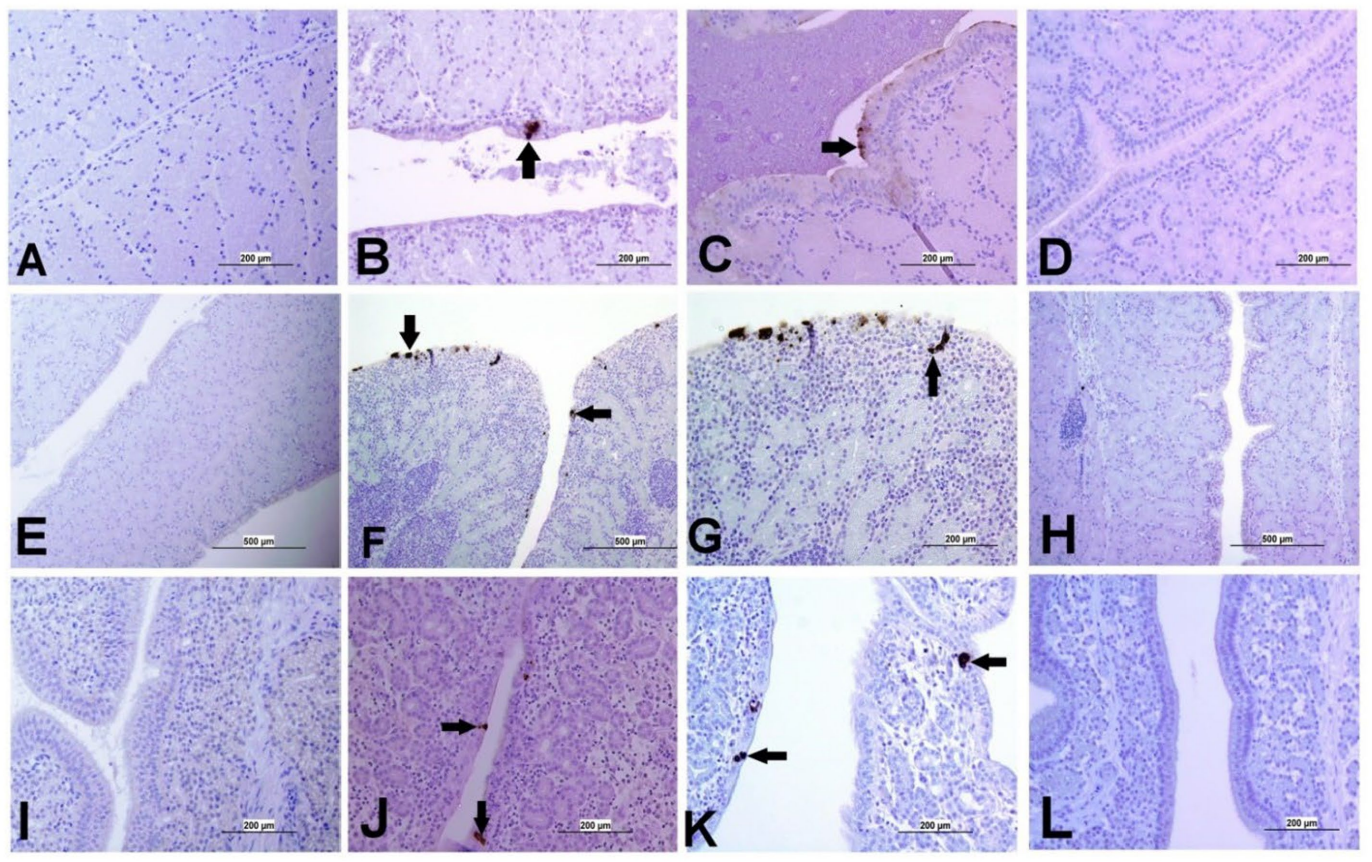
Representative images captured following immunohistochemical analysis of IBV antigens in different parts of oviduct at 21 dpi. **(A)** Magnum of control group showing no immuno-positive staining; **(B,C)** Magnum of DMV/1639 IBV-infected group showing positive viral antigen staining in epithelial lining (arrow). **(D)** Magnum of Mass IBV-infected group showing no immuno-positive reaction, **(E)** Isthmus of control group showing no immuno-positive staining; **(F,G)** Isthmus of DMV/1639 IBV-infected group showing strong immuno-positive reaction in epithelium and sub epithelium tissue (arrow). **(H)** Isthmus of Mass IBV-infected group with no immune-positive reaction. **(I)** Shell gland of control group showing no immuno-positive reaction. **(J,K)** Shell gland of DMV/1639 IBV-infected group; **(L)** Shell gland of Mass IBV-infected group with no immune-positive reaction.

**Figure 7:**
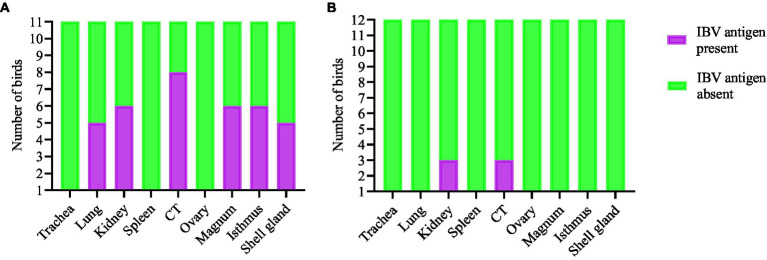
Expression of IBV antigen in different tissues through IHC sampled at 21 dpi. **(A)** Tissues from DMV/1639 IBV infected birds and **(B)** Tissues from Mass IBV infected birds.

The expression of IBV antigens in different tissues is presented in Figures 5, 6. At 21 dpi, no IBV viral antigen was observed in the control group and the tracheas, spleens, and ovaries of both infected groups. At the same time point, the viral antigen was observed in the lungs, kidneys, CT, and oviducts of the DMV/1639 IBV-infected group. The infected cells exhibited intracytoplasmic and finely to coarsely granular immune-positive reaction. In contrast, IBV antigen was detected only in the CT and kidney of three birds of the Mass IBV-infected group (Figure 7).

### 3.7 Gross pathology and egg quality

At 21 dpi, no macroscopic lesions were observed in the tissues of any bird from the control un-infected group. Marked gross pathology was observed in the oviduct of 5 out of 12 birds from the DMV/1639 infected group. Mainly, the lesions in the ovary were observed with distorted mature ova and egg peritonitis, whereas only 2 birds with congested ovary and oviduct were observed in the Mass-infected group (Supplementary Figure S1). No prominent gross pathology was observed in the other organs and tissues of both infected groups. Both the control and Mass-infected groups were producing eggs with normal internal and external qualities till the end of experiment. In contrast, 1 to 2 birds in the DMV/1639-infected group were producing deformed eggs with soft shell, thin shell, and meaty and bloody yolks from 8 dpi till the experiment was terminated (Supplementary Figure S1).

### 3.8 Histopathology

**Figure 8:**
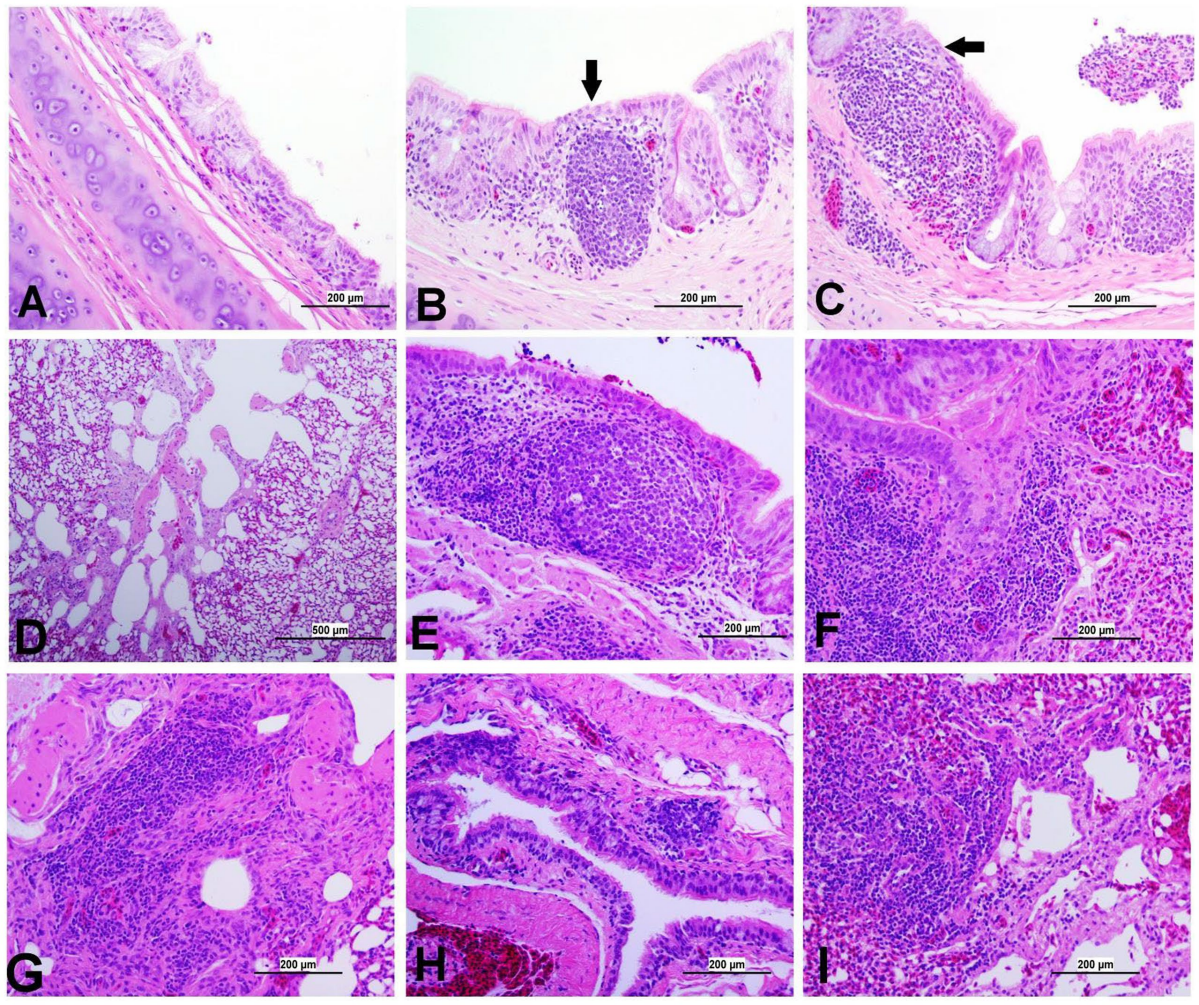
Representative photomicrographs of trachea and lungs of IBV-infected and control groups. **(A)** Trachea of control group; **(B)** Trachea of DMV1639 IBV-infected group showing focal area of epithelial degeneration and necrosis (arrow), mild mucosal mononuclear cell recruitment and lymphoid follicle aggregation. **(C)** Trachea of Mass IBV-infected group showing severe mucosal and submucosal mononuclear inflammatory cell and red blood cell aggregation, focal area of respiratory epithelial necrosis (arrow) and accumulation of mucus, cellular debris, inflammatory cells and red blood cells in the lumen. **(D)** Lung of control group; **(E,F)** Lung of DMV1639 IBV-infected group showing severe bronchitis. **(G)** Lung of DMV1639 IBV-infected group showing severe para bronchitis. **(H)** Lung of Mass IBV-infected group showing mild bronchitis. **(I)** Lung of Mass IBV-infected group showing moderate para bronchitis.

**Figure 9:**
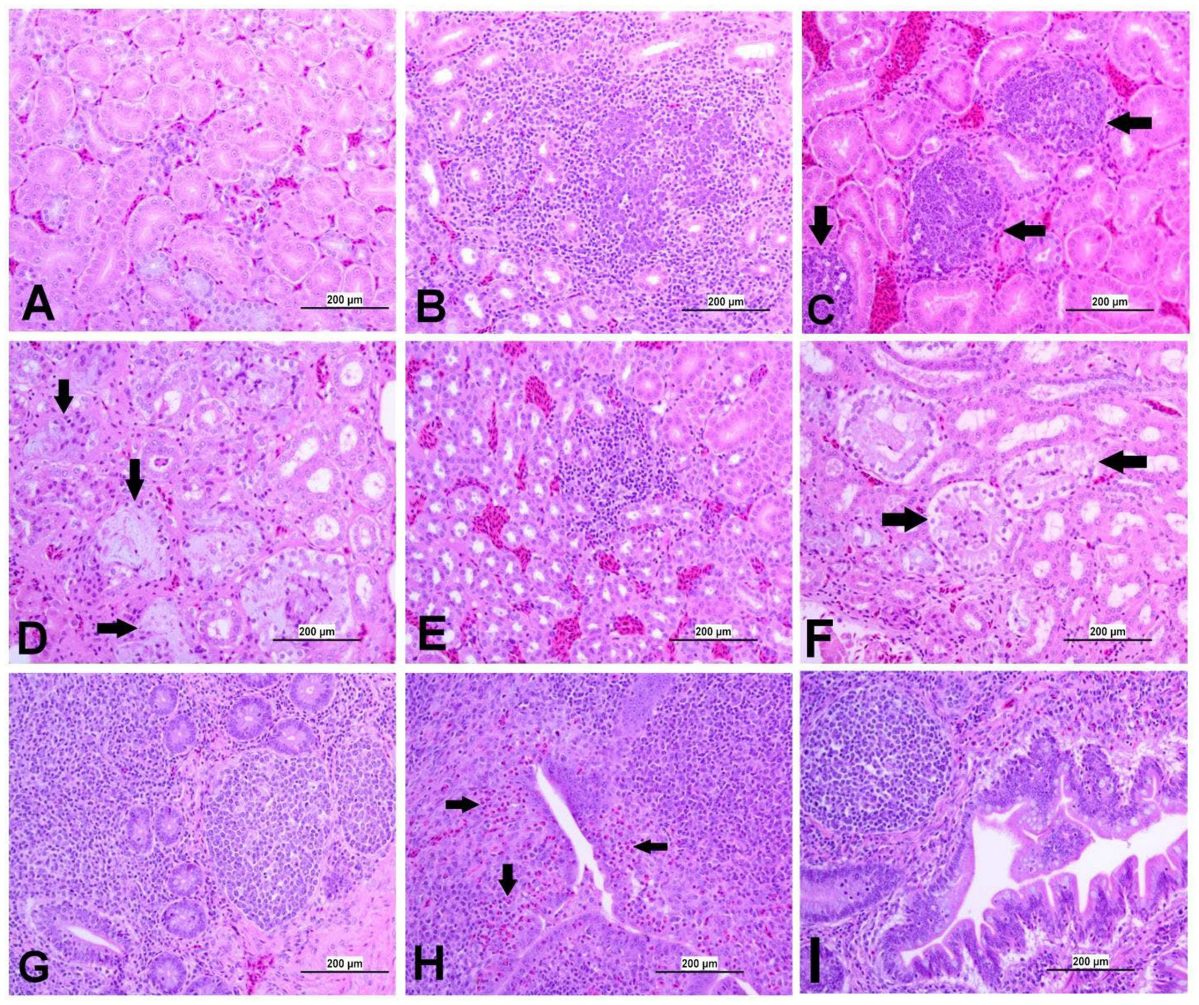
Representative photomicrographs of kidney and CT of IBV-infected and control groups. **(A)** Kidney of control group. **(B)** Kidney of DMV1639 IBV-infected group showing lymphoplasmacytic nephritis. **(C)** Kidney of DMV1639 IBV-infected group showing multiple interstitial lymphoid follicles aggregation (arrow). **(D)** Kidney of DMV1639 IBV-infected group showing marked degeneration and necrosis of epithelial lining renal tubules, some of them contain cellular debris, expansion of interstitial tissue with faint basophilic urates material surround by macrophages replacing normal renal tubules (arrow). and interstitial fibrosis. **(E)** Kidney of Mass IBV-infected group showing mild interstitial nephritis. **(F)** Kidney of Mass IBV-infected group showing moderate renal tubular necrosis with intraluminal accumulation of proteinaceous droplets and cellular debris (arrow). **(G)** CT of control group. **(H)** CT of DMV1639 IBV-infected group showing degeneration and single cell necrosis of lympho-epithelium, marked sub epithelial heterophilic infiltration (arrow) with intraluminal accumulation of cell debris, exudate and inflammatory cells; **(I)** CT of Mass IBV-infected group showing marked folding and hyperplasia of lympho-epithelium and Mucus cells with degeneration and necrosis of some of them, sub epithelial lymphocytes, macrophages and few heterophils aggregation.

**Figure 10:**
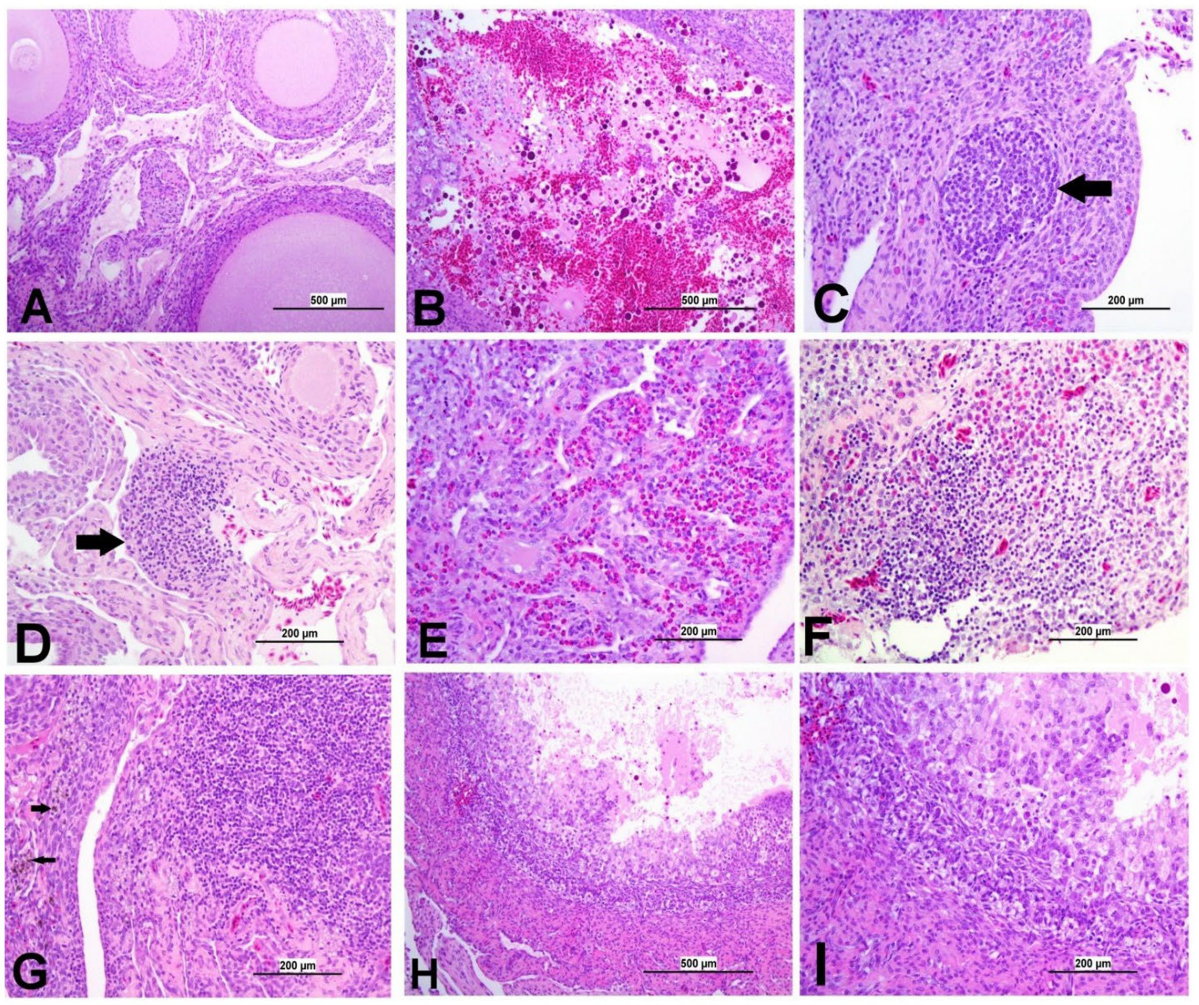
Representative photomicrographs of ovary of IBV-infected and control groups. **(A)** Ovary of control group showing normal histological findings. **(B)** DMV1639 IBV-infected group showing a large area of ovarian hemorrhage. **(C)** DMV1639 IBV-infected group showing lymphoid follicle aggregation (arrow). **(D)** DMV1639 IBV-infected group showing the focal area of mononuclear cell aggregation (arrow). **(E)** DMV1639 IBV-infected group showing diffuse heterophilic infiltration with few numbers of mononuclear cell aggregation. **(F)** Mass IBV-infected group showing diffuse mononuclear cell infiltration with heterophilic aggregation. **(G)** Mass IBV-infected group showing the large focal area of lymphocytic aggregation with an accumulation of hemosiderin-laden macrophages (arrow). **(H)** Mass IBV-infected group showing obliterative follicular atresia. **(I)** Higher magnification of the previous image **(H)** shows indistinct perivitelline membrane, invasion of granulosa, and theca interna cells filling the follicle and destroying the oocyte with inflammatory cell aggregation in the follicular wall.

**Figure 11:**
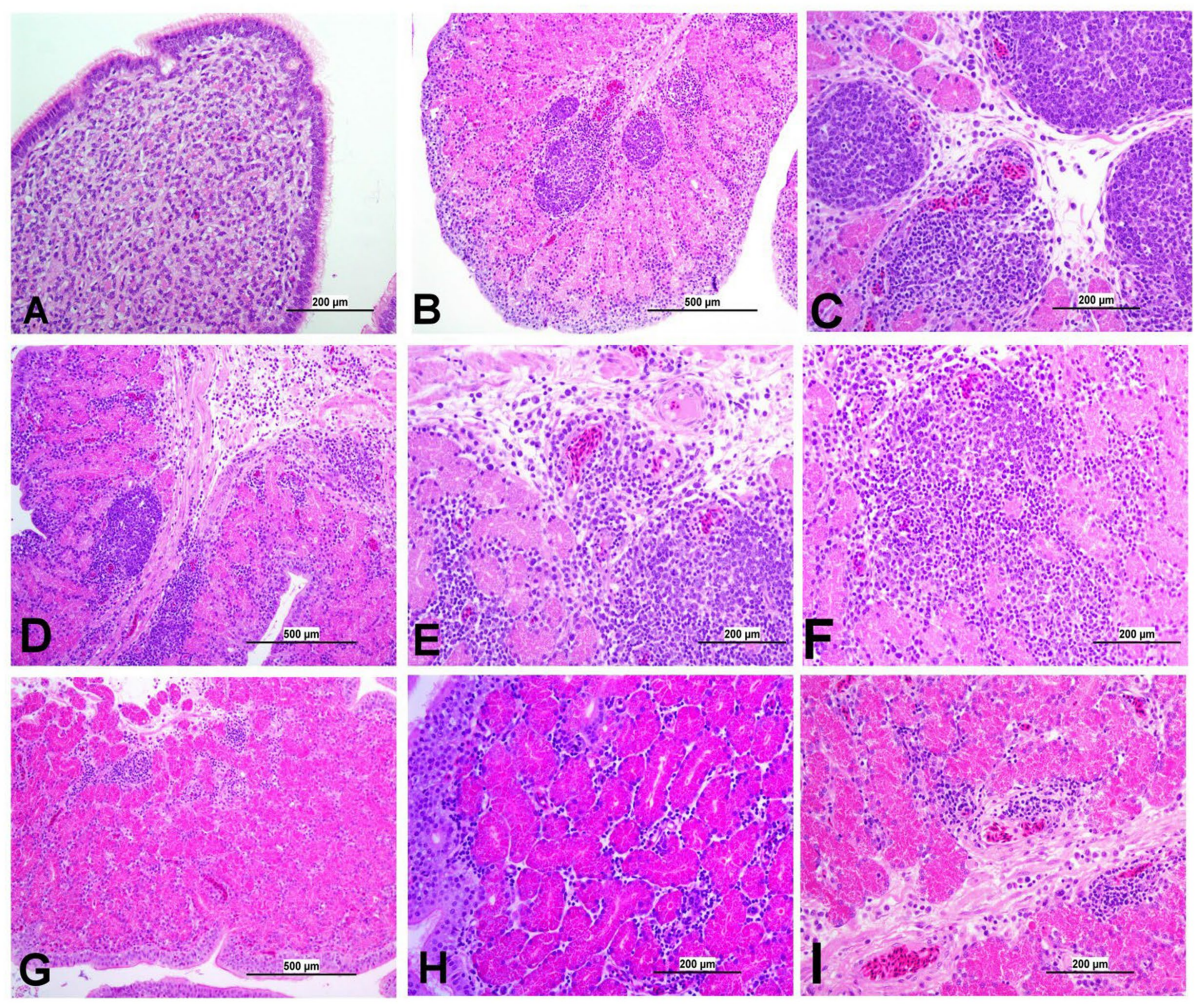
Representative photomicrographs of Isthmus of IBV-infected and control groups. **(A)** Isthmus of control group; **(B)** DMV1639 IBV-infected group showing severe salpingitis. **(C)** DMV1639 IBV-infected group showing marked perivascular edema with lymphoplasmacytic aggregation and multiple lymphoid aggregations. **(D)** DMV1639 IBV-infected group showing marked mucosal and muscular mononuclear inflammatory cell infiltration. **(E,F)** DMV1639 IBV-infected group showing glandular necrosis which replaced by large number of lymphoplasmacytic cells. **(G)** Mass IBV-infected group showing moderate salpingitis. **(H)** Mass IBV-infected group showing hyperplasia of mucosal epithelium, glandular necrosis and mononuclear cell aggregation. **(I)** Mass IBV-infected group showing mucosal congestion, mild perivascular mononuclear cell aggregation, mild glandular necrosis and mucosal mononuclear cell aggregation.

**Figure 12:**
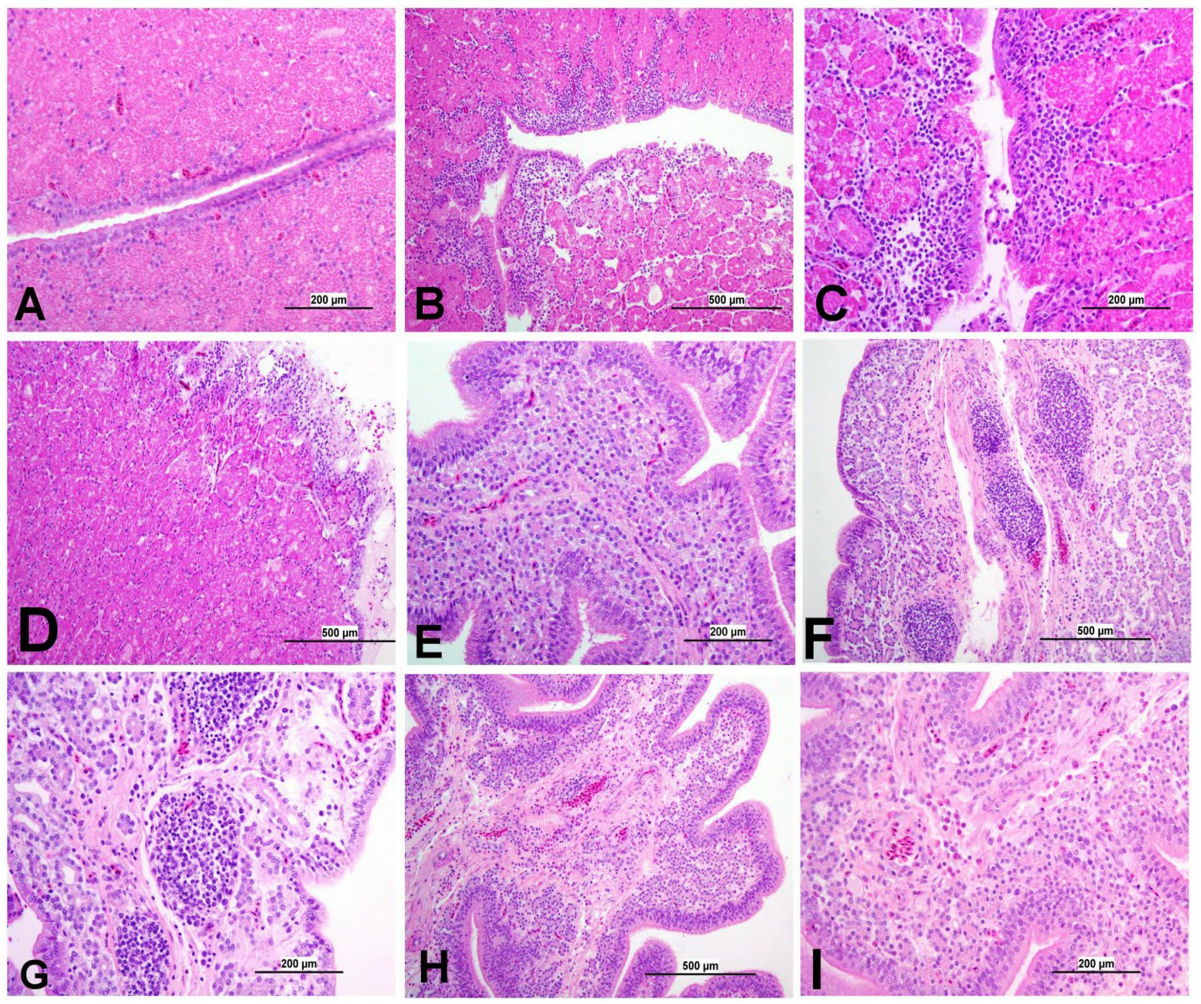
Representative photomicrographs of magnum and shell gland of IBV-infected and control groups. **(A)** Magnum of control group; **(B,C)** Magnum of DMV1639 IBV-infected group showing degenerative changes and necrosis in mucosal epithelium and secretory glands along with lamina propria infiltration with mononuclear cells and accumulation of cellular debris and inflammatory cells in the lumen. **(D)** Magnum of Mass IBV-infected group showing marked degeneration, necrosis of mucosal epithelium, mononuclear inflammatory cell aggregations in epithelium and sub-epithelium and mild secretory gland necrosis. **(E)** Shell gland of control group; **(F,G)** Shell gland of DMV1639 IBV-infected group showing multiple mucosal lymphoplasmacytic aggregations, epithelial and glandular necrosis. **(H,I)** Shell gland of Mass IBV-infected group showing edema, mild glandular necrosis and inflammatory cell aggregation mainly heterophils and lymphocytes.

**Figure 13:**
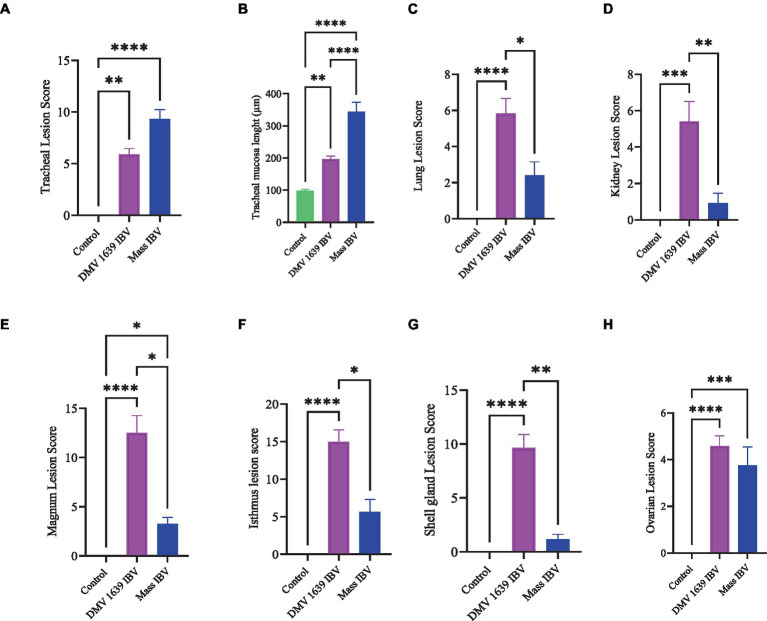
Lesion scores of different organs of IBV-infected and mock-infected groups. **(A)** Tracheal lesion scores. **(B)** Tracheal mucosal thickness. **(C)** Lung lesion scores. **(D)** Kidney lesion scores. **(E)** Magnum lesion scores. **(F)** Isthmus lesion scores. **(G)** Shell gland lesion scores. **(H)** Ovarian lesion scores. Values expressed as median with interquartile range and were analyzed using the Kruskal-Wallis test followed by Dunn’s multiple comparison. Significance: *p < 0.05, **p < 0.01, ***p < 0.001.

At 21 dpi, no histopathological lesions were observed in the trachea, lung, kidney, spleen, CT, ovary, and oviduct of the control group. No obvious histopathological changes were observed in the spleen of both IBV-infected groups. The lesion observed in different organs is presented in Figures 8–12, providing quantitative data in Figure 13. The DMV/1639 IBV-infected group exhibited the most severe lesions in multiple organs, whereas the lesions observed in the Mass IBV-infected group were comparatively less severe.

#### 3.8.1 Lesions in the trachea

The study results revealed a clear contrast between the control group and the two IBV-infected groups. While the trachea of the control group was in a healthy state, both IBV-exposed groups exhibited histopathological lesions. Of the two infected groups, the one exposed to Mass IBV displayed more severe lesions and thicker mucosal tissues than the group infected with DMV/1639-IBV. The observed lesions in the IBV-infected trachea include loss of epithelium in some areas, degeneration and necrosis of epithelial lining trachea, depletion of mucus glands, and marked diffuse mucosal and submucosal lymphoplasmacytic infiltration with marked lymphoid follicles hyperplasia in mucosa, while some areas are lined by regenerative hyperplastic epithelial cells (Figures 8A–C).

#### 3.8.2 Lesions in the lung

The lung of the control group was normal. The nature of the lesion was the same in both IBV-infected groups, but the DMV/1639 IBV-infected group showed more severe lesions. Primary and secondary bronchi showed inflammation with marked folding and hyperplasia of its epithelial lining, mucus accumulation in the bronchial lumen, and massive mononuclear cell recruitment in lamina propria with marked lymphoid follicle aggregation. The parabronchial septa were thickened with edema, congestion, and inflammatory cell aggregation (Figures 8E–I).

#### 3.8.3 Lesions in the kidney

The kidneys of the control group were normal (Figure 9A). Most of birds infected with DMV/1639 IBV strain showed moderate to severe and focal to diffuse lymphocyte infiltration in the interstitial tissue with dilation and necrosis of epithelial lining renal tubules. Some renal tubules contained cellular cast and urate material. In addition, lymphoid follicle generation was observed in many birds. In contrast, the Mass IBV-infected group revealed mild medullary lymphocytic interstitial nephritis with significant reduction in lesion score (Figure 13) when compared with the DMV/1639 IBV-infected group (Figures 9B–F).

#### 3.8.4 Lesions in the cecal tonsils

The CT of the control group was normal. The DMV/1639 IBV-infected group revealed degeneration and necrosis of lymphoepithelial lining of CT with aggregation of heterophils and foamy macrophages in subepithelial zone and interfollicular area, while hyperplasia of epithelial lining and mucus cells was observed in the Mass IBV-infected group. Mild lymphoid depletion was detected in both IBV-infected groups (Figures 9G–I).

#### 3.8.5 Lesions in the ovary

The ovary of the birds in the control group was normal, as shown in Figure 10A. The ovarian lesions were more severe in the DMV/1639 IBV-infected group when compared with the Mass IBV-infected group. The lesions were present as cystic atresia, characterized by breakdown of yolk, loss of the perivitelline membrane definition, marked vacuolar degeneration of the granulosa cells, ovarian congestion, hemorrhage, and heterophilic infiltration with lymphocytic oophoritis. In addition, the Mass IBV-infected group revealed aggregation of some lymphoid follicles (Figures 10B–I).

#### 3.8.6 Lesions in the oviduct

The oviduct of the control group was normal, as shown in Figures 11, 12. The lesions were mild to moderate in the Mass IBV-infected group and moderate to severe in the DMV/1639 IBV-infected group. The isthmus was a part of the oviduct, which was affected the most in both groups (Figure 11). The magnum of the DMV/1639 IBV-infected group showed moderate-to-severe alterations in the form of epithelial degeneration and necrosis with subepithelial mononuclear cell aggregation, necrosis of the gland, marked expansion of the lamina propria with mononuclear inflammatory cells, perivascular lymphocytic cuffing, and mononuclear cell aggregation in the muscular layer (Figures 12B–D). The shell gland of the DMV/1639 IBV-infected group exhibited degeneration of epithelial lining, necrosis of some glands, interstitial edema, focal mononuclear cell aggregation in lamina propria with marked perivascular edema, and lymphocytic cuffing. On the other hand, the uterus of the Mass IBV-infected group showed mild-to-moderate lesion in some birds, and there was a significant difference in lesion scoring between the two IBV-infected groups (Figures 11–13).

### 3.9 TUNEL assay for apoptosis detection in tissue sections

**Figure 14:**
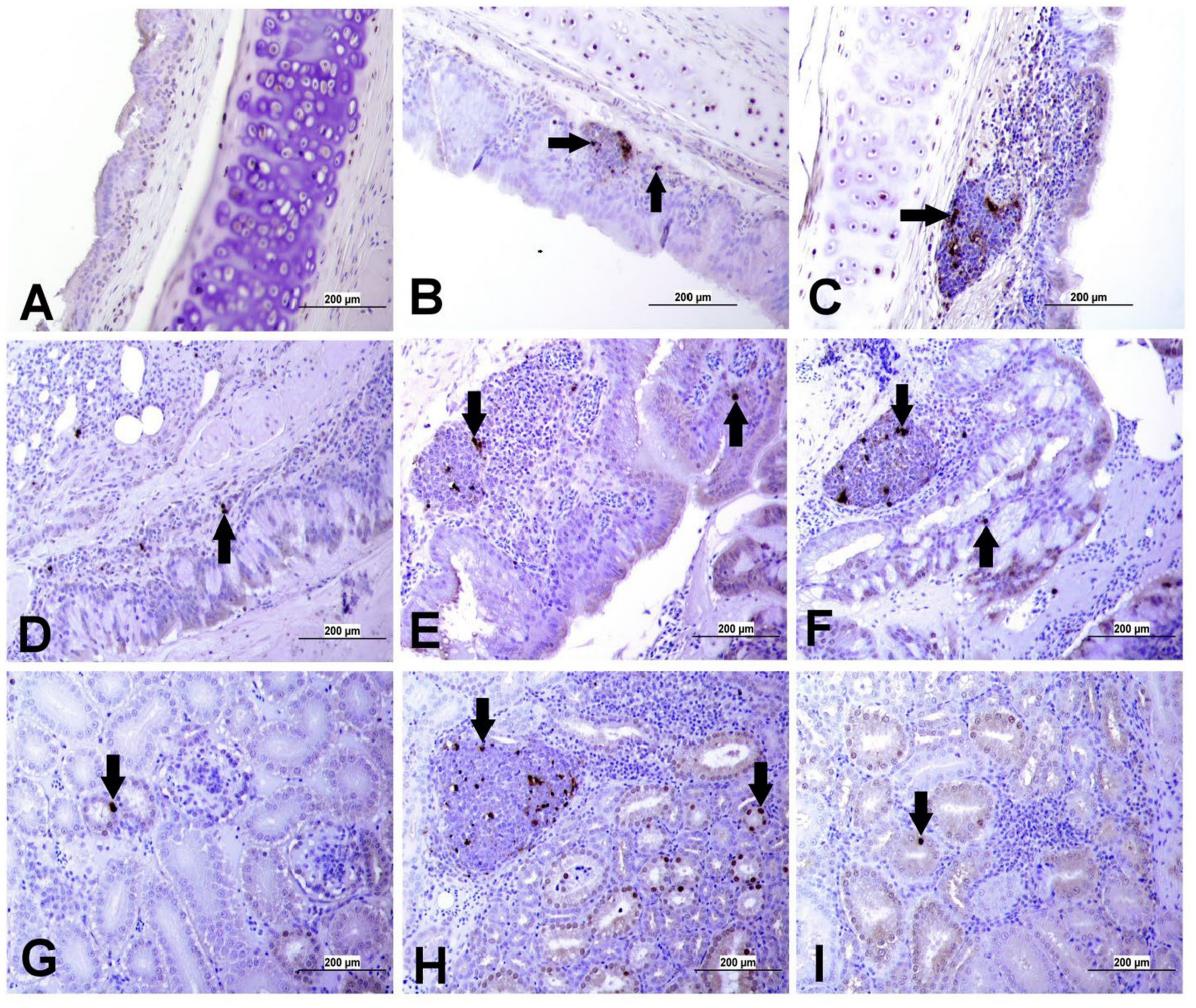
Apoptosis detection using Tunnel assay in the trachea, lung and kidney of different groups. Black arrow indicates Tunnel positive cell. **(A)** Trachea of the control group **(B)** Trachea of IBV DMV1639 infected group showing apoptotic cells in mononuclear inflammatory cells. **(C)** Trachea of Mass group showing apoptotic cells in lymphoid follicle and mononuclear inflammatory cells. **(D)** Lung of control group. **(E)** Lung of IBV DMV1639 infected and **(F)** Lung of Mass IBV infected group showing apoptotic cells in lymphoid follicle, mononuclear inflammatory cells and epithelial lining bronchi. **(G)** Kidney of control group. **(H)** Kidney of IBV DMV1639 infected group showing Tunnel positive cells in lymphoid follicle, and epithelial lining renal tubules. **(I)** Kidney of Mass group showing Tunnel positive cell in epithelial lining renal tubules.

**Figure 15:**
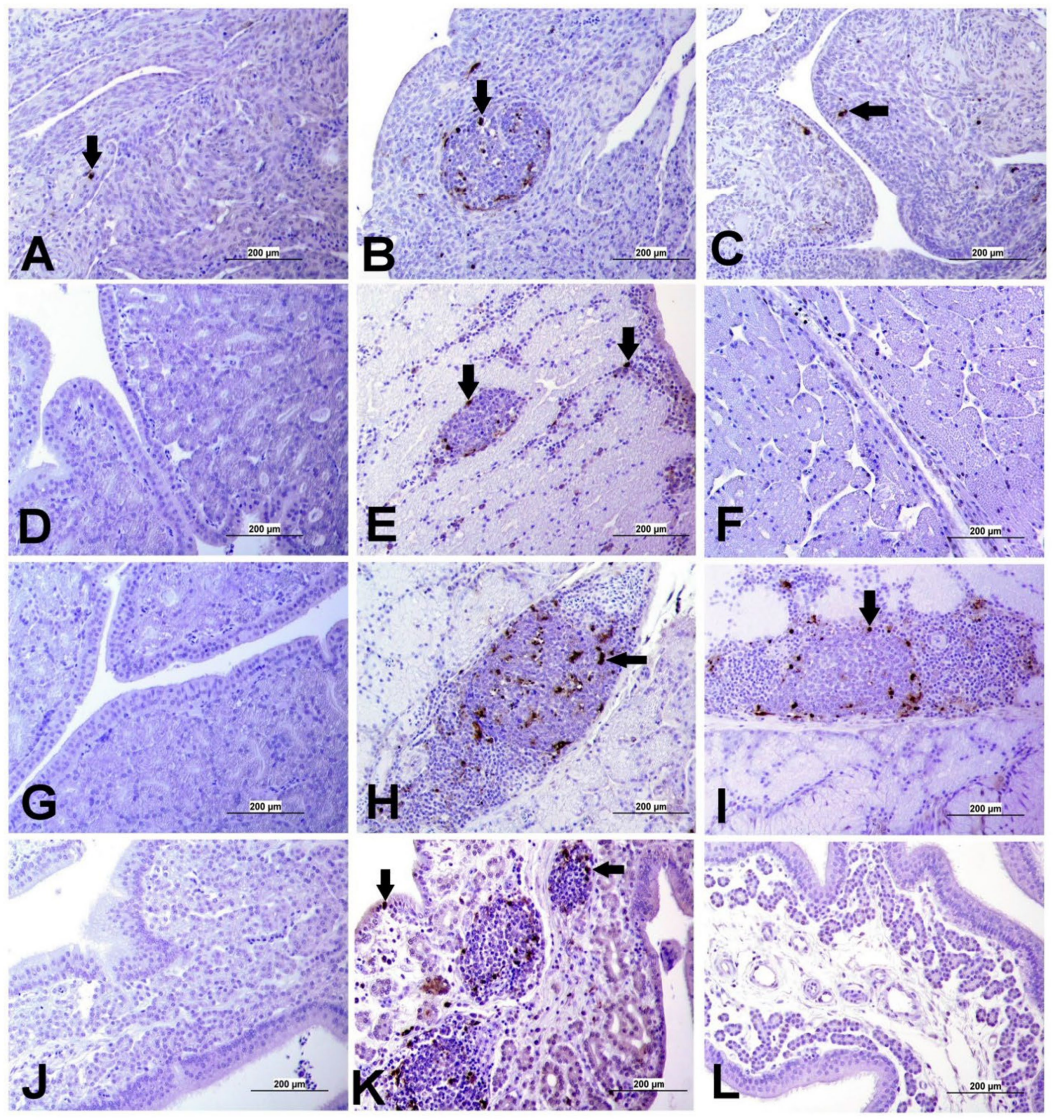
Apoptosis detection using Tunnel assay in ovary and oviduct of different groups. Black arrow indicates Tunnel positive cell. **(A)** Ovary of control group **(B)** Ovary of IBV DMV1639 infected group showing positive cells in lymphoid follicles aggregates and stromal cells. **(C)** Ovary of Mass IBV infected group. **(D)** Magnum of control group. **(E)** Magnum of IBV DMV1639 infected group. **(F)** Magnum of Mass IBV infected group. **(G)** Isthmus of control and **(H)** Isthmus of IBV DMV1639 infected groups showing positive cells in lymphoid follicle, and mononuclear inflammatory cells. **(I)** Isthmus of Mass group. **(J)** Shell gland of control group. **(K)** Shell gland of IBV DMV1639 infected group showing Tunnel positive cells in lymphoid follicles aggregates, mononuclear inflammatory cells and epithelial shell gland. **(L)** Shell gland of Mass IBV infected group.

**Figure 16:**
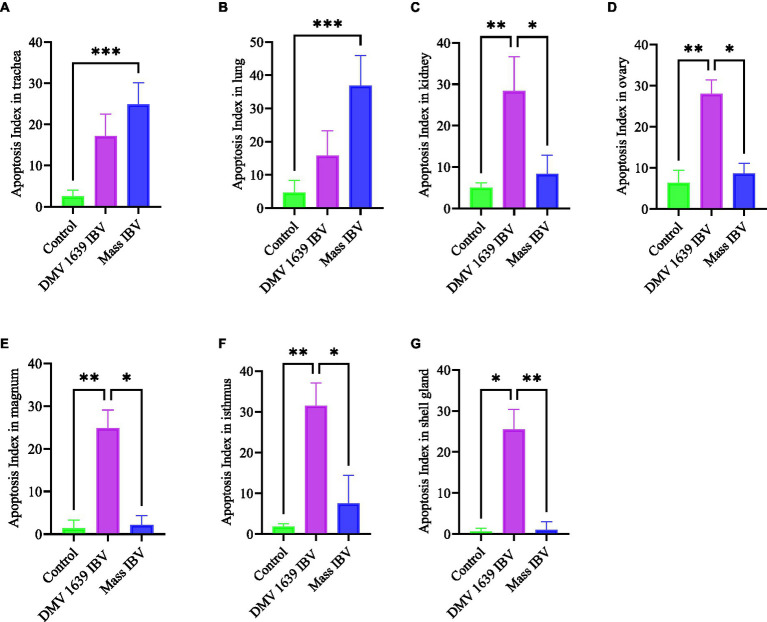
Bar chart represented the apoptosis Index of different organs. **(A)** Trachea. **(B)** Lung. **(C)** Kidney. **(D)** Ovary. **(E)** Magnum. **(F)** Isthmus. **(G)** Shell gland. Values expressed as mean + − SE and were analyzed using the Kruskal-Wallis test followed by Dunn’s multiple comparison test. Significance: *p < 0.05, **p < 0.01, ***p < 0.001.

Apoptotic cells were detected in all three experimental groups. The apoptotic cells had a dark brown nucleus. The apoptotic cells were normally observed in the epithelium lining trachea, primary and secondary bronchi, renal tubular epithelium, ovarian stromal cells, the epithelium lining atretic follicles, and lining epithelium of the magnum, isthmus, and shell gland, as shown in Figures 14, 15. However, both IBV DMV1639 and Mass groups revealed no significant difference in the apoptosis index in the trachea and lungs. Concerning the kidney, the DMV1639 infection showed an increase in the apoptosis index compared with the control and Mass IBV-infected groups. The positive apoptotic cells were observed in renal tubules, lymphoplasmacytic cells, and lymphoid follicle aggregates (Figures 14H,I). In the ovary, magnum, isthmus, and shell gland, there were significant elevations in the apoptosis index in the DMV1639-infected group when compared with the Mass and control groups. Many apoptotic cells were detected in mononuclear inflammatory cell aggregates, lymphoid follicle aggregates, and epithelial lining ovary and oviduct (Figure 15). The apoptotic index of cells in different tissues is presented in Figure 16.

## 4 Discussion

This experiment was designed to compare the pathogenicity of two IBV isolates belonging to two major genotypes, Mass and DMV/1639. In the Eastern Canada, DMV/1639 IBV is prevalent (22, 31), and in the Western Canada, Mass IBV is prevalent (8). Our study yielded three significant findings. First, we observed a significant drop in egg numbers in hens infected with DMV/1639 IBV. This suggests that the DMV/1639 IBV strain is more pathogenic in laying hens compared with the Mass IBV strain. Second, we observed that virus-induced pathology is significant in tissues (except the trachea) of chickens infected with DMV/1639 IBV than the Mass IBV-infected chickens. Third, we observed that Mass IBV induced significantly higher apoptosis rates in the respiratory tissues, whereas DMV/1639 IBV was able to induce higher apoptosis rates in the renal and oviduct tissues.

The appearance of clinical signs followed by dropped egg production in this study is consistent with previous studies on IBV infection in SPF layers (7). In this study, it has been observed that the onset of clinical signs, including decline in egg numbers on 2 and 5 dpi, respectively, in mature hens, is infected with the DMV/1639 strain of IBV. Even though the infection of laying hens with DMV/1639 IBV in our study was in line with previous observations, we did not find a significant drop in egg numbers in hens due to Mass IBV infection, unlike earlier studies (8). It is difficult to explain this inconsistency in egg production in Mass IBV-infected laying hens between current and previous studies; it is possible that differences between housing system and low number of animals in the previous study may have influenced the infection in the reproductive system, leading to discrepancy in egg production data. We raised the birds (n = 12 birds in the pen) in floor pens, providing adequate space, perching, and nest boxes, and the previous study has been performed in high containment isolators with limited space (n = 3 in the space of 4 × 4′ cubicles.) However, further studies are necessary to rule out these possibilities in observed discrepancy in egg production data, following Mass IBV infection in laying hens.

Poor internal egg quality, following IBV infection, has been recorded previously (32, 33). The thick albumin height of both DMV/1639 IBV-infected and Mass IBV-infected groups was significantly (*p* < 0.05) less than the control group post-infection. IBV infection typically effects the cells of the magnum, leading to thin and watery albumin which ultimately reduces the Haugh unit values (34). Pathological changes in the glandular epithelial cells of the tubular glands in the magnum may result in the reduced synthesis of albumin protein (35). In the current study, we observed that both DMV/1639 IBV and Mass IBV-infected groups induced histological changes in the magnum 21 dpi, coinciding with IBV replication at least in the DMV/1639 IBV-infected group.

Several experimental animal models investigating apoptosis demonstrate a strong relationship between the rate of apoptosis and the virus infection (14), and this agrees with the current observation. In *ex vivo* and *in vitro* conditions, it has been shown that Mass IBV induces greater apoptosis in tracheal organ culture than in kidney cell culture, and nephropathogenic IBV induces greater apoptosis in the kidney than in the tracheal organ culture. The induction of cell apoptosis by viruses may lead to tissue damage (36), and this was reflected in the pathology-induced Mass IBV, which is more severe in the trachea and DM1639 IBV induced pathology, which is more severe in the kidney.

In conclusion, both DMV/1639 and Mass IBV isolates are capable of infecting mature laying hens at their peak of lay, causing severe clinical signs, such as respiratory distress and decreased egg production. Although both strains are virulent, the DMV/1639 strain of IBV has a wider range of tissue tropism and a particular affinity for the kidney and oviduct of laying hens, leading to severe damage and a significant drop in egg production. The Mass IBV strain primarily targets the respiratory tract of laying hens, causing respiratory distress. The severity and extent of apoptosis varied between the DMV/1639 and Mass IBV strains, with the former strain causing more extensive apoptosis in the kidneys and oviducts, while the latter primarily affected the tracheal epithelium. The results highlight the importance of strain-specific differences in IBV pathogenicity and their potential impact on the poultry industry.

## Data availability statement

The raw data supporting the conclusions of this article will be made available by the authors, without undue reservation.

## Ethics statement

The animal study was approved by Veterinary Science Animal Care Committee (VSACC) of the University of Calgary (AC19-0011). The study was conducted in accordance with the local legislation and institutional requirements.

## Author contributions

MF: Investigation, Methodology, Validation, Writing – review & editing, Formal Analysis, Visualization, Writing – original draft. RA-E: Formal Analysis, Investigation, Methodology, Validation, Visualization, Writing – original draft, Writing – review & editing. NR: Formal Analysis, Investigation, Writing – review & editing. MH: Formal Analysis, Investigation, Visualization, Writing – review & editing. SN: Formal Analysis, Investigation, Visualization, Writing – review & editing. SCo: Methodology, Supervision, Validation, Visualization, Writing – review & editing. SCh: Methodology, Supervision, Validation, Visualization, Writing – review & editing. YN: Methodology, Supervision, Validation, Visualization, Writing – review & editing. MA-C: Conceptualization, Funding acquisition, Investigation, Methodology, Project administration, Resources, Supervision, Validation, Writing – review & editing.
