# Supplementary material for: Comparative pathogenicity of infectious bronchitis virus Massachusetts and Delmarva (DMV/1639) genotypes in laying hens
Source: Front Vet Sci. 2024 Jan 19;10:1329430. doi: 10.3389/fvets.2023.1329430 (PMC10834656; doi:10.3389/fvets.2023.1329430)
Supplement: Supplementary file 1 [file Table_1.docx]

**Supplementary Figures**


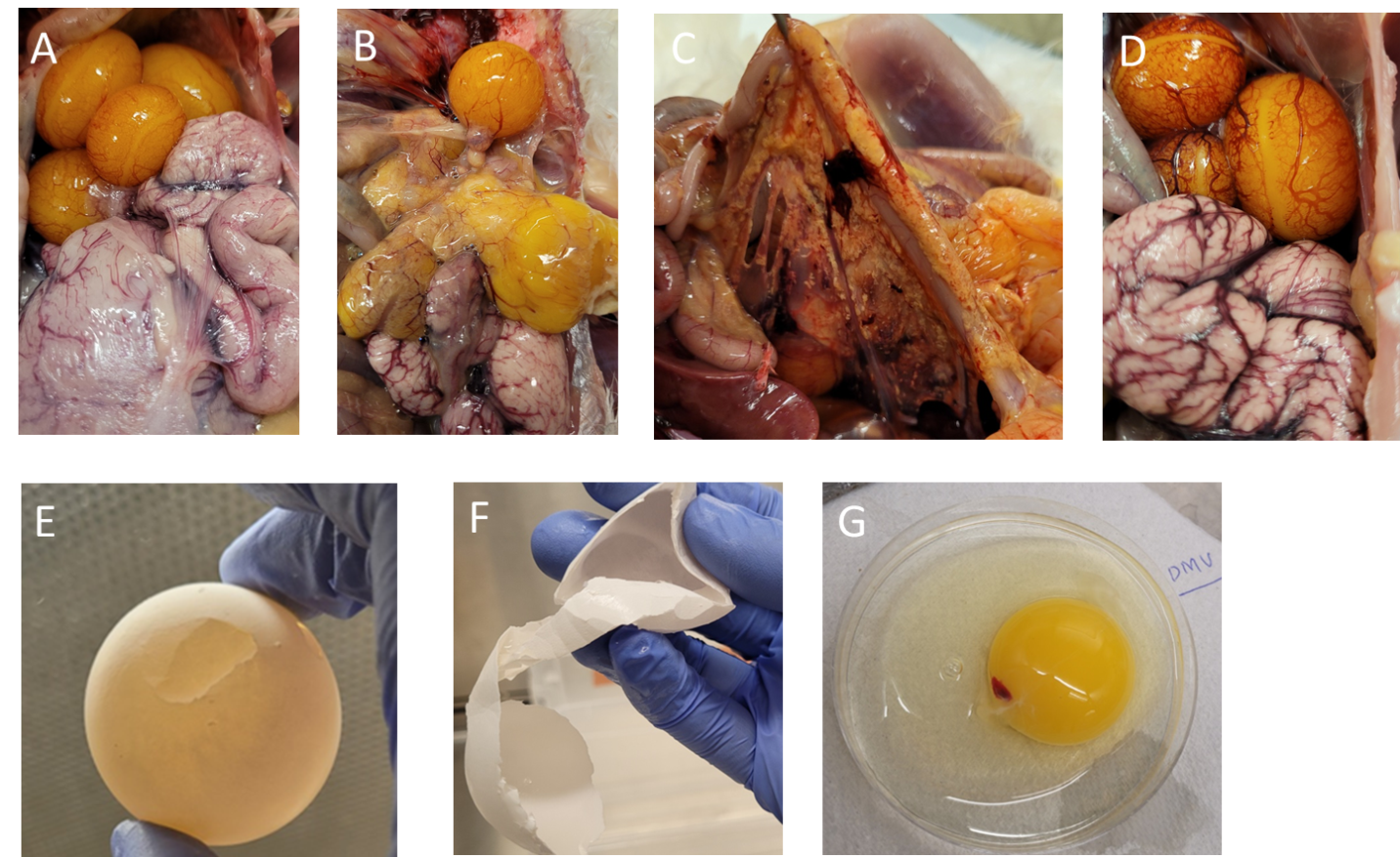


Supplementary Figure 1. Images of gross lesions in the ovary, reproductive tract, external and internal egg quality. (A) Ovary and oviduct of the control group (B) distorted yolks in the ovary of the DMV/1639 infected group (C) Egg peritonitis in the DMV/1639 infected group (D) Congested ovary and oviduct in Mass infected group (E-F) Soft shell eggs in the 1639/DMV infected group (G) Meaty and bloody yolk in the DMV/1639 infected group.
