# Supplementary material for: Comparative pathogenicity of infectious bronchitis virus Massachusetts and Delmarva (DMV/1639) genotypes in laying hens
Source: Front Vet Sci. 2024 Jan 19;10:1329430. doi: 10.3389/fvets.2023.1329430 (PMC10834656; doi:10.3389/fvets.2023.1329430)
Supplement: Supplementary file 2 [file Data_Sheet_1.docx]

**Supplementary Figures**


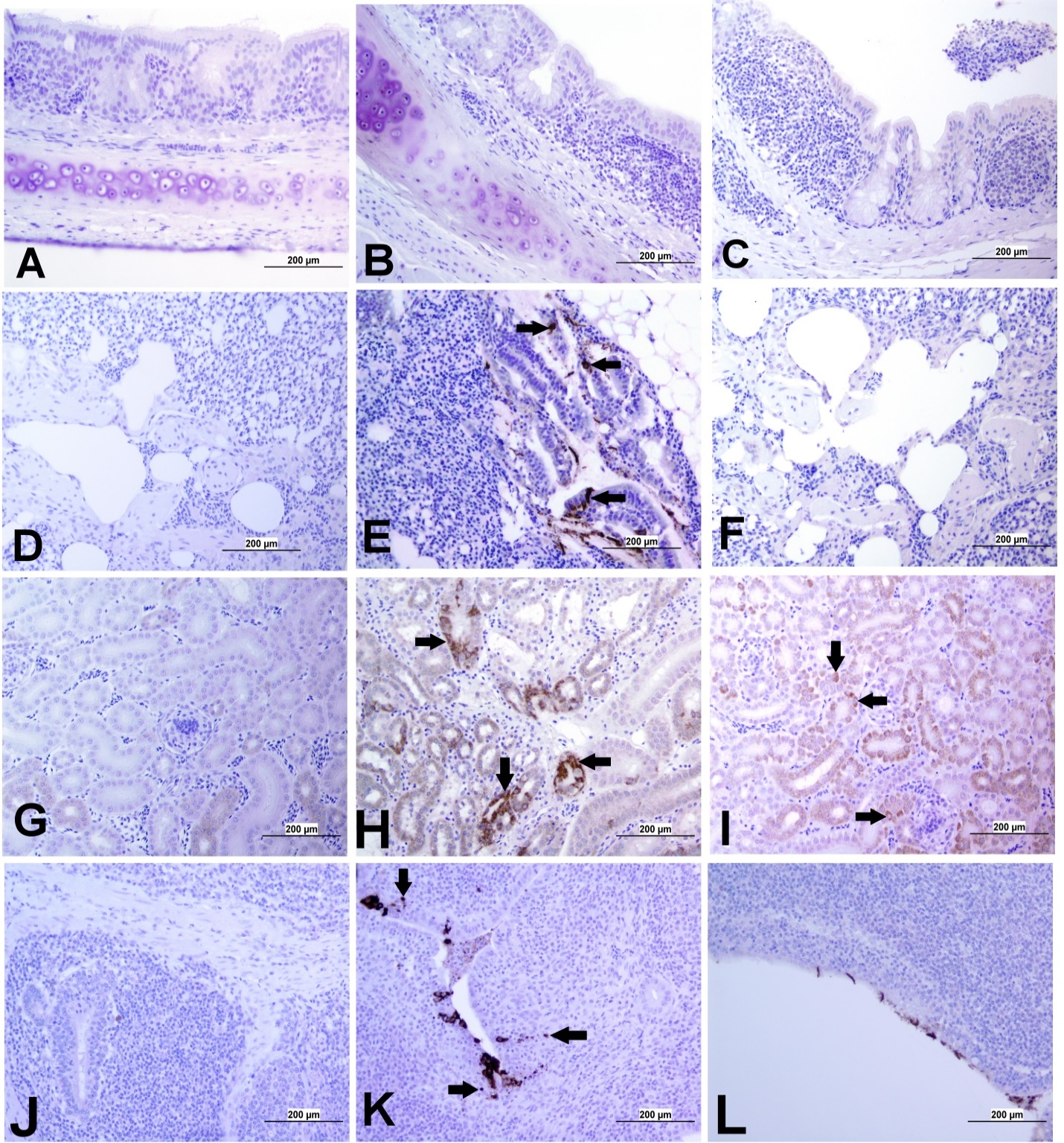


Supplementary Figure 1. Representative images captured following immunohistochemical analysis of IBV antigens in different tissues at 21 dpi. (A) Trachea of control group, (B) Trachea of DMV/1639 IBV-infected group and (C) Trachea of Mass IBV-infected group showing no immuno-positive reaction. (D) Lung of control group; (E) Lung of DMV/1639 IBV-infected group showing immuno-positive staining in epithelium lining of bronchi (arrow). (F) Lung of Mass IBV-infected group showing no immuno-positive reaction. (G) Kidney of control group; (H) Kidney of DMV/1639 IBV-infected group showing strong immune-positive reaction in renal tubules (arrow). (I) Kidney of Mass IBV-infected group showing weak immuno-positive reaction in renal tubules (arrow). (J) CT of control group; (K) CT of DMV/1639 IBV-infected group showing strong immuno-positive reaction in lymphoepithelium and sub-epithelium inflammatory cells (arrow); (L) CT of Mass IBV-infected group with immuno-positive reaction in the
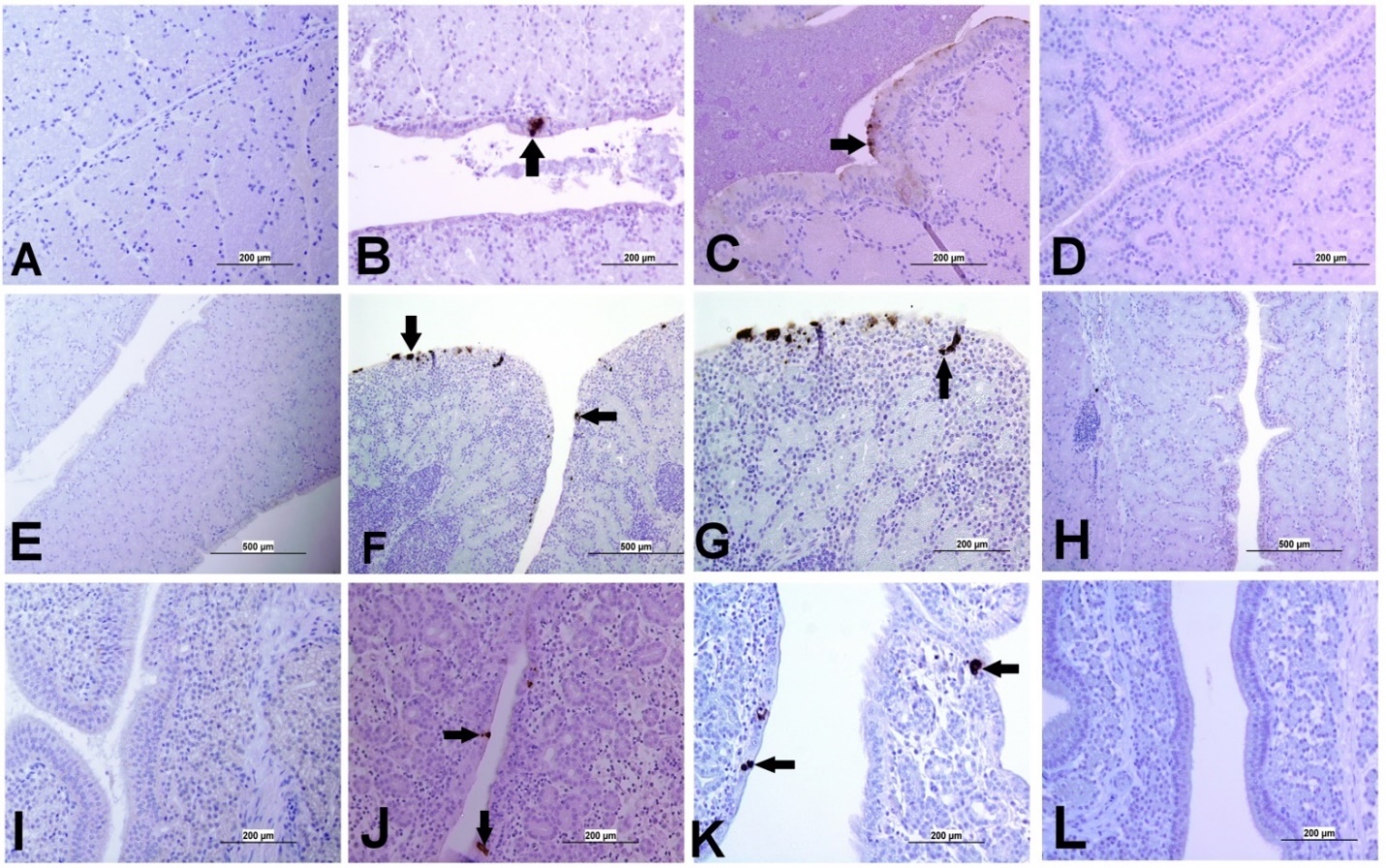
epithelium.

Supplementary Figure 2. Representative images captured following immunohistochemical analysis of IBV antigens in different parts of oviduct at 21 dpi. (A) Magnum of control group showing no immuno-positive staining; (B-C) Magnum of DMV/1639 IBV-infected group showing positive viral antigen staining in epithelial lining (arrow). (D) Magnum of Mass IBV-infected group showing no immuno-positive reaction, (E) Isthmus of control group showing no immuno-positive staining; (F-G) Isthmus of DMV/1639 IBV-infected group showing strong immuno-positive reaction in epithelium and sub epithelium tissue (arrow). (H) Isthmus of Mass IBV-infected group with no immune-positive reaction. (I) Shell gland of control group showing no immuno-positive reaction. (J-K) Shell gland of DMV/1639 IBV-infected group; (L) Shell gland of Mass IBV-infected group with no immune-positive reaction.

Supplementary Figure 3. Expression of IBV antigen in different tissues through IHC sampled at 21 dpi. (A) Tissues from DMV/1639 IBV infected birds and (B) Tissues from Mass IBV infected birds.


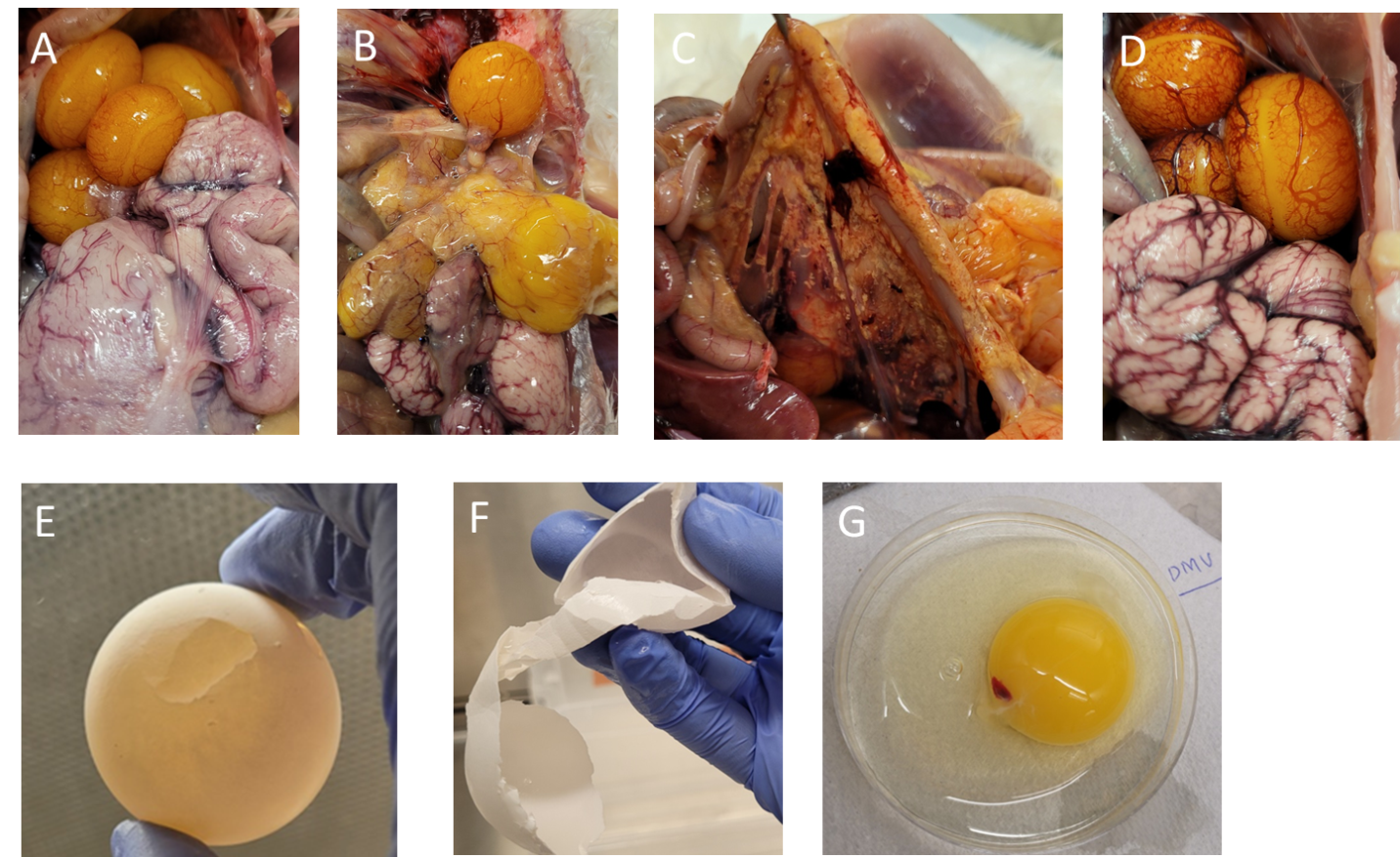


Supplementary Figure 4. Images of gross lesions in the ovary, reproductive tract, external and internal egg quality. (A) Ovary and oviduct of the control group (B) distorted yolks in the ovary of the DMV/1639 infected group (C) Egg peritonitis in the DMV/1639 infected group (D) Congested ovary and oviduct in Mass infected group (E-F) Soft shell eggs in the 1639/DMV infected group (G) Meaty and bloody yolk in the DMV/1639 infected group.
